# Gestational Diabetes Mellitus and Infant Adiposity at Birth: A Systematic Review and Meta-Analysis of Therapeutic Interventions

**DOI:** 10.3390/jcm10040835

**Published:** 2021-02-18

**Authors:** Manoja P. Herath, Jeffrey M. Beckett, Andrew P. Hills, Nuala M. Byrne, Kiran D. K. Ahuja

**Affiliations:** School of Health Sciences, College of Health and Medicine, University of Tasmania, Launceston, TAS 7248, Australia; manoja.herath@utas.edu.au (M.P.H.); jeffrey.beckett@utas.edu.au (J.M.B.); andrew.hills@utas.edu.au (A.P.H.); nuala.byrne@utas.edu.au (N.M.B.)

**Keywords:** gestational diabetes mellitus, treatment, adiposity, fat mass, skinfold thickness, newborns, infants

## Abstract

Exposure to untreated gestational diabetes mellitus (GDM) in utero increases the risk of obesity and type 2 diabetes in adulthood, and increased adiposity in GDM-exposed infants is suggested as a plausible mediator of this increased risk of later-life metabolic disorders. Evidence is equivocal regarding the impact of good glycaemic control in GDM mothers on infant adiposity at birth. We systematically reviewed studies reporting fat mass (FM), percent fat mass (%FM) and skinfold thicknesses (SFT) at birth in infants of mothers with GDM controlled with therapeutic interventions (IGDMtr). While treating GDM lowered FM in newborns compared to no treatment, there was no difference in FM and SFT according to the type of treatment (insulin, metformin, glyburide). IGDMtr had higher overall adiposity (mean difference, 95% confidence interval) measured with FM (68.46 g, 29.91 to 107.01) and %FM (1.98%, 0.54 to 3.42) but similar subcutaneous adiposity measured with SFT, compared to infants exposed to normal glucose tolerance (INGT). This suggests that IGDMtr may be characterised by excess fat accrual in internal adipose tissue. Given that intra-abdominal adiposity is a major risk factor for metabolic disorders, future studies should distinguish adipose tissue distribution of IGDMtr and INGT.

## 1. Introduction

The prevalence of gestational diabetes mellitus (GDM) is rising globally, affecting up to 38% of pregnancies in some populations [1]. As well as causing complications during pregnancy and delivery including macrosomia, shoulder dystocia and preterm birth, exposure to GDM in utero places offspring at an increased risk of obesity and type 2 diabetes in later life [2,3]. The mechanisms associated with this increased risk of obesity and type 2 diabetes are not well understood; however, increased adiposity during foetal growth has been suggested as a potential mediator [4]. The Pedersen hypothesis [5] suggests that, as glucose freely crosses the placenta, maternal hyperglycaemia in diabetic pregnancies leads to foetal hyperinsulinaemia, causing accelerated foetal uptake of glucose (foetal glucose steal phenomenon) and deposition of excess foetal adipose tissue [6]. The impact of GDM on adipose tissue growth in the foetus can be identified with adiposity measures at birth, for example, fat mass (FM), percent fat mass (%FM) and skinfold thickness (SFT) [7].

Diagnosis and management of GDM continue to be controversial. The earlier definition of GDM, i.e., “any degree of glucose intolerance that occurs or is first diagnosed during pregnancy” [8], was used for many years and enabled a uniform approach to the detection of GDM. However, the classification of women with unrecognized overt diabetes as GDM and providing treatments accordingly may not be effective because risks associated with type 1 and type 2 diabetes are greater than GDM [9]. In the latest clinical practice recommendations by the American Diabetes Association [10], GDM is defined as “glucose intolerance first diagnosed during the second or third trimester of pregnancy in women without overt diabetes prior to pregnancy, which resolves postnatally”, and this involves risk-based screening for type 2 diabetes or prediabetes at their initial prenatal visit. Nonetheless, different criteria are currently being used worldwide to diagnose GDM. A landmark change in these diagnostic thresholds occurred when the Hyperglycaemia and Adverse Pregnancy Outcome (HAPO) study [11] demonstrated a positive linear association between increasing levels of plasma glucose and adverse pregnancy outcomes and subsequently, lowered thresholds for screening GDM. These new diagnostic thresholds (fasting plasma glucose 5.1–6.9 mmol/L, 1-h plasma glucose  ≥ 10.0 mmol/L or 2-h 8.5–11.0 mmol/L) were promulgated by the International Association of Diabetes and Pregnancy Study Groups (IADPSG) in 2010 and by the World Health Organisation (WHO) in 2013, and this enabled detection of more GDM cases [12].

Awareness of the adverse outcomes associated with GDM has been a driver for substantial improvements in perinatal care for pregnant women with GDM in recent years [13]. The first-line treatment for GDM involves lifestyle changes, e.g., modified diet and increased physical activity, and nearly two-thirds of women can achieve glycaemic targets with this approach [14]. When blood glucose levels are not adequately controlled with modified lifestyle alone, supplementary pharmacological treatments such as metformin, glyburide or insulin are added to the therapeutic regimen [15]. Glycaemic control in GDM women using modified dietary interventions alone has resulted in lower birth weights and less macrosomia [16], despite the high heterogeneity in diet observed among different populations [17]. Similarly, using pharmaceutical interventions along with or without lifestyle changes has resulted in reduced risk of macrosomia [18] and has prevented GDM-associated adverse health conditions in neonates [19]. Nevertheless, the effect of GDM treatments on neonatal adiposity is understudied, and the evidence for whether good glycaemic control in GDM can normalise foetal adiposity is contradictory [20,21,22]. To ascertain the impact of glycaemic control in GDM on infant adiposity at birth, we systematically reviewed studies reporting adiposity in newborns of mothers with GDM controlled with therapeutic interventions.

## 2. Materials and Methods

This work was conducted following the Preferred Reporting Items for Systematic Reviews and Meta-Analyses (PRISMA) guidelines [23]. Our protocol is registered in PROSPERO (CRD42020175338).

### 2.1. Search Strategy

Electronic searches were conducted in three stages with the assistance of a Research Librarian at the University of Tasmania, Australia. First, a limited search was undertaken in Medline and Scopus, using search terms: “gestational diabetes”, “body composition” and “infants”. The title, abstract and index terms of the retrieved articles were scanned to build a keyword list. In the second step, a broader search was conducted (March 2020), using the identified terms in MEDLINE in Ovid, Embase, CINHAL, PubMed and Web of Science databases, limiting the results to studies published in “English” language, “human” species and “infants” age group. The search strategy for MEDLINE is shown in Figure S1, and a similar approach was used in other databases. Finally, we manually scanned the reference lists of included articles, relevant reviews, and citations to identify any additional studies. Hand searches were not conducted for any specific journal, and we did not trace any grey literature.

### 2.2. Eligibility Criteria

We included all study types reporting adiposity in infants exposed to GDM. Inclusion criteria were: (1) data collected at birth or <1-month infants’ age; (2) availability of infant adiposity measure(s); i.e., fat mass (FM), percent fat mass (%FM) or skinfold thickness (SFT); and (3) availability of information regarding what therapeutic measures were undertaken to control GDM. Exclusion criteria were (1) examination of maternal glycaemia as a continuous variable; (2) assessment of only foetal measurements (e.g., ultrasound scans); (3) merging of data for GDM exposed infants with pregestational diabetes-exposed infants; (4) full report of the study not published in English; and (5) review articles, protocol papers and conference abstracts. When there were multiple publications from the same sample of study participants, we only included the paper that presented the most appropriate data for the purpose of this review.

### 2.3. Study Selection

The results emanating from database searches were imported into the Covidence software^®^ [24]. After removing duplicates, the search outputs were independently reviewed at the title and abstract level by M.P.H. and K.D.K.A./J.M.B. to find potentially eligible articles. These articles were screened at the full-text level by the same reviewers to determine the eligibility of the papers for data extraction.

### 2.4. Quality Assessment

The methodological quality of the selected studies was assessed by two reviewers (M.P.H. and J.M.B) using the Evidence Project risk of bias tool. This tool is appropriate for assessing study rigour for both randomised and non-randomised intervention studies. The Evidence Project risk of bias tool includes eight items: (1) cohort, (2) control or comparison group, (3) pre-post intervention data, (4) random assignment of participants to the intervention, (5) random selection of participants for assessment, (6) follow-up rate of 80% or more, (7) comparison groups equivalent on sociodemographics, and (8) comparison groups equivalent at baseline on outcome measures [25]. For criterion 7, we considered infant sex and ethnicity as the relevant sociodemographic characteristics. If authors reported that study arms were equivalent on only one sociodemographic variable, we considered the meeting of the criterion as “Partial”. Additionally, if the study arms were not equivalent on at least one sociodemographic variable, we considered that the criterion was not met. Any disagreements between the two reviewers regarding the inclusion of studies and quality assessment were resolved by discussion and consensus.

### 2.5. Data Extraction

A pre-designed data collection form was used to extract information from each paper. This information included: (1) study characteristics (author, year, design, time period of data collection, state/country of the study, inclusion and exclusion criteria), (2) study groups (sample size, male%), (3) method(s) (GDM screening/diagnostic criteria, treatments to control GDM, target blood glucose level, degree of glycaemic control, body composition measurement technique) and (4) outcomes (FM, %FM, SFT).

### 2.6. Data Analysis

For the purpose of data synthesis, the included studies were categorised according to comparison groups: (1) ‘treated’ GDM vs. ‘untreated’ GDM; (2) different treatment regimens for GDM and, (3) treated GDM vs. normal glucose tolerance (NGT). When blood glucose levels of GDM mothers were controlled with any form of therapeutic intervention (including lifestyle modification and/or pharmaceutical interventions), they were considered as ‘treated’, and usual antenatal care without any specific treatment for GDM was considered as ‘untreated’. When an adequate number of studies were available, meta-analyses were performed with the inverse variance statistical method and random effects analysis model (RevMan version: 5.4.0) [26]. Mean difference at a 95% confidence interval was used to combine the results. Forest plots were used to demonstrate the outcomes. Heterogeneity between the studies in meta-analyses was determined with a Chi^2^ test on the Q statistic (variance of the observed effect sizes in the meta-analysis), Tau^2^ (between-study variance of the true effect sizes) and I^2^ (proportion of the observed variation in the effect size due to differences in the true underlying effect sizes, as opposed to sampling error). An alpha level <0.05 was considered statistically significant. Potential sources of heterogeneity, i.e., level of glycaemic control in GDM mothers, any advances in the effectiveness of treatments for GDM in ‘recent’ years (defined as study data collection occurred during or after 2010: referred as post-2010) compared to ‘pre-2010’ (defined as study data collection occurred before 2010), GDM diagnosis criteria and body composition assessment technique, were investigated with subgroup analyses. Sensitivity testing was performed with ‘leave-one-out’ testing.

## 3. Results

### 3.1. Study Selection

**Figure 1:**
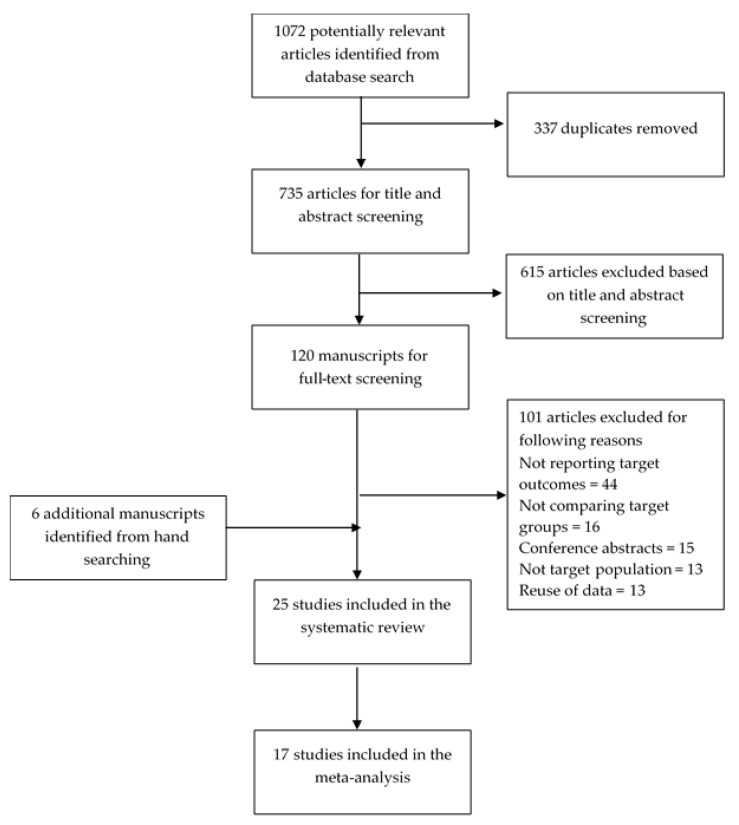
Flow of information through the different phases of the review.

Of the 1072 references identified through database searching, 19 matched inclusion-exclusion criteria (Figure 1). An additional six papers were identified through a review of reference lists, relevant reviews, and forward citations. In total, 25 studies [7,20,21,22,27,28,29,30,31,32,33,34,35,36,37,38,39,40,41,42,43,44,45,46,47] were included in the systematic review, of which 17 [7,20,21,22,27,28,29,31,33,37,38,39,41,42,43,44,45] were included in the meta-analysis.

### 3.2. Description of the Studies

The selected references included three randomised clinical trials [34,35,36], 15 cohort studies [7,21,27,29,30,31,37,38,41,42,43,44,45,46,47], 3 case-control studies [20,33,39,42] and 4 cross-sectional studies [22,28,32,40]. The included studies were published between 1980 and 2020, and from 12 different countries, i.e., the United States [7,28,29,31,35,36,40], Australia [21,22,45], New Zealand [30,33], Australia and New Zealand [34], Germany [37,38,41], Sweden [20,44], China [32], France [43], Italy [27], Malaysia [47], Spain [39], the United Kingdom [46] and Turkey [42]. Sample sizes varied from 25 to 1000 (Table 1).

Eleven guidelines developed between 1964 and 2014 were used for screening and diagnosing GDM by 22 studies (Table S1). The remaining three studies [20,33,47] used centre-specific criteria for screening and diagnosing GDM. From the 25 studies, 24 used an oral glucose tolerance test (OGTT) to diagnose GDM, and the other [17] used White’s classification based on the age of onset and duration of diabetes. Commonly used criteria included Carpenter and Coustan (1982), Australasian Diabetes in Pregnancy Society (ADIPS, 1998), and the International Association of Diabetes and Pregnancy Study Groups (IADPSG, 2011). Seven of the guidelines utilised a screening oral glucose challenge test (OGCT) prior to an OGTT, while other guidelines used only a diagnostic OGTT. Only six of the guidelines tested plasma glucose 3-h post OGCT. The cut-offs for fasting, 1-h, 2-h and 3-h blood glucose ranged between 5.0–7.0 mmol/L, 9.2–11.0 mmol/L, 8.0–9.1 mmol/L and 6.9–8.0 mmol/L, respectively. Seven GDM criteria required two or more abnormal values, while three guidelines required only one abnormal value, for diagnosis of GDM.

Fasting and 2-h post-prandial plasma glucose targets for treated-GDM mothers differed between studies as follows; 5.0 mmol/L and 6.7 mmol/L [33,37], 5.3 mmol/L and 7.8 mmol/L [29], 5.3 mmol/L and 6.7 mmol/L [25,26], 5.5 mmol/L and 6.5 mmol/L [23], 5.5 mmol/L and 6.7 mmol/L [6,19], and 5.5 mmol/L and 7.0 mmol/L [11,12,20,24]. Two studies [10,32] used HbA1c between 3.5–5.3% as the mean blood glucose target.

#### 3.2.1. Treatments Used to Control GDM and Level of Glycaemic Control

Three studies [17,18,22] used ‘diet only’, and one study [23] used ‘insulin only’ to treat GDM while others used combinations of treatments such as ‘diet with insulin if required’ [10,11,19,20,21,26,27,28,29,31,33,37], ‘diet and exercise with insulin, if required’ [6,12,32,34,36]. Metformin, alone or in combination with insulin, was used in two studies [24,35], while glyburide alone or in combination with diet was used in another two studies [25,30]. Only 60% of the studies (*n =* 15) reported the level of glycaemic control in GDM women.

#### 3.2.2. Adiposity Assessment Techniques Used in the Studies

Anthropometric and/or body composition information was available in 13 studies, including air displacement plethysmography (ADP) [11,12,30,34], total body electrical conductivity (TOBEC) [6,21], or anthropometric equations proposed by Catalano et al. [10,27,35,37], Dauncy et al. [17,29], and Weststrate and Deurenberg [31]. The most commonly assessed individual SFT sites were triceps and subscapular, and four studies [17,31,33,37] presented the sum of SFT at different sites (data of individual sites were not available).

### 3.3. Quality Assessment

Of the eight criteria listed in the Evidence Project risk of bias tool, two criteria, “(3) pre-post intervention data”, and “(8) comparison groups equivalent at baseline on outcome measures”, were not applicable for the studies selected for this review (Table 2). All selected studies used non-probability sampling strategies (convenience or self-selected sampling); thus, the criterion “random selection of participants for assessment” was not met by any of them. All studies met the “control or comparison group” criterion. Nineteen studies [6,11,17,19,20,21,24,25,26,27,28,29,31,32,33,34,35,36,37] met the criterion “cohort”, and except for 1 study [20], all others met the criterion of “follow-up rate of 80% or more”. Only the three randomised control trials (RCT) [24,25,26] included in the review met the criterion of “(4) random assignment of participants to the intervention”. Results of the assessment of the criterion “(7) comparison groups equivalent on sociodemographics” varied across the studies, with the following outcomes: “Equivalent” [20,23,25,30], “Partially Equivalent” [6,21,22,24,26,27,28,29,32,34,36], “Not Equivalent” [12,31,37], and “Not Reported” [10,11,17,18,19,33,35].

### 3.4. Effects of Treatments for GDM on Infant Adiposity

#### 3.4.1. Treated GDM vs. No Treatment for GDM

One RCT [36] investigated whether treatment for GDM normalised infant adiposity at birth. In this study of 958 GDM women (485 treated vs. 473 no treatment), mean FM in infants of GDM mothers who received the treatment of diet therapy (*n =* 427) and insulin, if required (*n =* 36), was significantly lower (*p =* 0.003) than that of control infants whose mothers received usual prenatal care (427 ± 198 g vs. 464 ± 222 g).

#### 3.4.2. Different Treatment Regimens for GDM

Two studies [7,30] compared the effects of treating GDM with lifestyle modification alone vs. lifestyle modification plus insulin, on infant birth measurements. A small study [30] with a sample size of 20, found no significant differences in mean subscapular SFT, between GDM exposed infants whose mothers were treated with ‘diet alone’ and ‘diet with insulin’. A comparatively larger study [7], with a sample size of 195, revealed that compared to ‘diet and exercise only’, infants whose mothers were treated with ‘diet, exercise and insulin’ had higher FM (492 ± 215 g vs. 407 ± 196 g, *p =* 0.006) and %BF (13.6% ± 4.6% vs. 11.7 ± 4.5%, *p =* 0.007). These effects persisted even after adjusting for gestational age, maternal pregravid weight and parity. Two RCTs [34,35] investigated the difference in adiposity in infants of GDM mothers, who were treated with pharmacological treatments for GDM. Rowan et al. [34] compared treating GDM women with metformin (with supplemental insulin, if required, *n =* 363) to treatment with insulin alone (*n =* 370) and reported that triceps (5.2 ± 1.6 vs. 5.1 ± 1.2, *p =* 0.30) and subscapular (5.2 ± 1.5 vs. 5.2 ± 1.3, *p =* 0.60) SFT (mm) were not significantly different between the groups. Lain et al. [35] compared insulin (*n =* 41) with glyburide (*n =* 41), and found no significant differences in mean triceps SFT (3.9 ± 0.7 vs. 3.9 ± 0.9, *p =* 0.89), subscapular SFT (4.1 ± 1.0 vs. 4.5 ± 1.3, *p =* 0.10), suprailiac SFT (2.1 ± 0.6 vs. 2.1 ± 0.6, *p =* 0.85), thigh SFT (5.1 ± 1.2 vs. 5.4 ± 1.7, *p =* 0.28), FM (370 ± 167 vs. 473 ± 278, *p =* 0.06) or %FM (11.2 ± 4.2 vs. 12.8 ± 5.7, *p =* 0.18). None of the studies compared ‘lifestyle modification alone’ with ‘pharmaceutical interventions’.

#### 3.4.3. Treated GDM vs. NGT

##### Fat Mass (FM)

**Figure 2:**
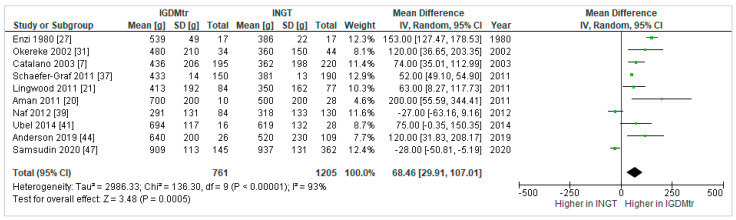
Forest plot comparing fat mass (g) in infants exposed to treated gestational diabetes mellitus (IGDMtr) and infants exposed to normal glucose tolerance (INGT).

Ten studies [7,20,21,27,31,37,39,41,44,47] reported the effect of treated GDM compared to NGT on infant FM. Overall, infants born to mothers with treated GDM (IGDMtr) had significantly higher FM (mean difference, 95% confidence interval: 68.46 g, 29.91 to 107.01) than infants born to NGT mothers (INGT); (Figure 2).

##### Percent Fat Mass (%FM)

**Figure 3:**
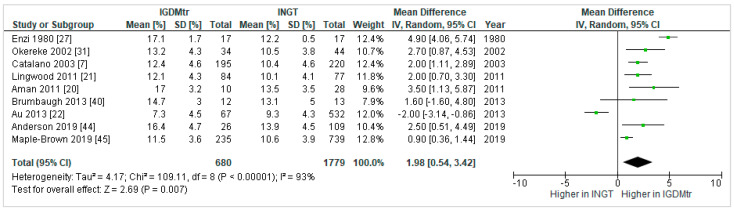
Forest plot comparing percent fat mass (%) in infants exposed to treated gestational diabetes mellitus (IGDMtr) and infants exposed to normal glucose tolerance (INGT).

Nine studies [7,20,21,22,27,31,40,44,45] investigated the effect of treated GDM compared to NGT on infant %FM. In the pooled result, %FM (1.98%, 0.54 to 3.42) in IGDMtr was significantly higher than INGT (Figure 3).

##### Skinfold Thickness (SFT)

**Figure 4:**
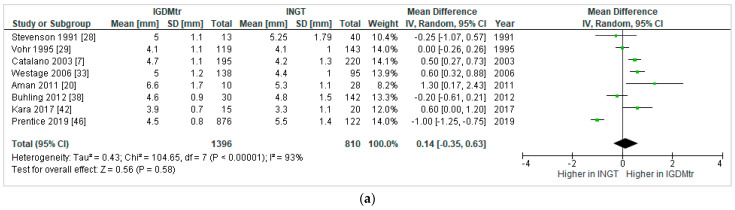

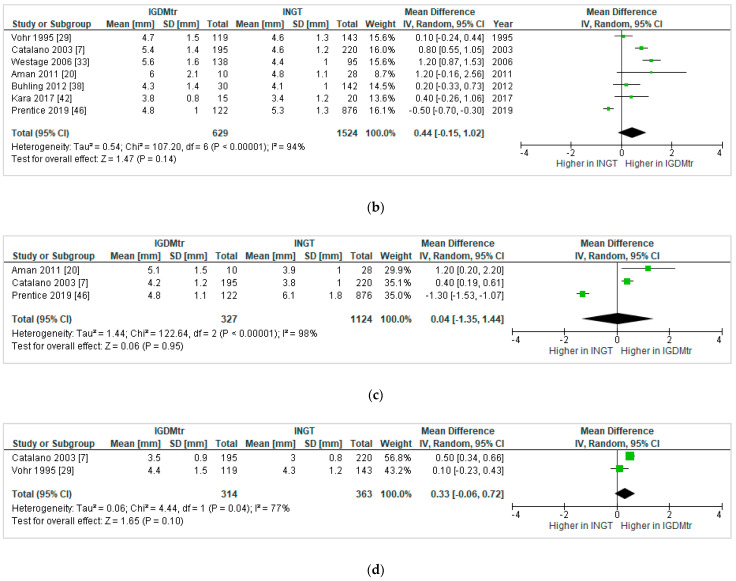
Forest plots comparing skinfold thickness (mm) at triceps (**a**), subscapular (**b**), flank (**c**) and abdomen (**d**) in infants exposed to treated gestational diabetes mellitus (IGDMtr) and infants exposed to normal glucose tolerance (INGT).

The number of studies that reported SFT at individual skinfold sites were as follows: triceps *=* 8 [7,20,28,29,33,38,42,46]; subscapular *=* 7 [7,20,29,33,38,42,46]; flank *=* 3 [7,20,46]; and abdominal *=* 2 [7,29]. None of the comparisons of skinfold sites were significantly different between IGDMtr and INGT infants in the pooled results; triceps: 0.14 mm, −0.35 to 0.63 (Figure 4a); subscapular: 0.44 mm, −0.15 to 1.02 (Figure 4b); flank: 0.04 mm, −1.35 to 1.44 (Figure 4c) and abdominal: 0.33 mm, −0.06 to 0.72 (Figure 4d). Several other SFT sites, i.e., biceps [42], quadriceps [46], suprailiac [29], iliac [38], femur [38], thigh [7] and calf [29], were reported in single studies, and therefore, a meta-analysis could not be performed.

Four studies compared IGDMtr vs. INGT using sum of SFT at different body sites, therefore they were not included in the meta-analysis. Of those, two reported that the sum of SFT at ‘triceps and subscapular’ [40] and ‘subscapular, subcostal, tricipital and crural’ [27] was significantly higher in IGDMtr. Another study [47] reported that the sum of SFT at ‘flank, triceps and subscapular’ was not significantly different between IGDMtr and INGT. A study [32] in which data were not normally distributed presented median and interquartile range and reported that triceps (4.8 mm (4.2–5.1) vs. 4.7 mm (4.1–5.5)) and subscapular (4.8 mm (4.3–5.3) vs. 4.8 mm (4.1–5.3)) SFT were not significantly different between the two infant groups.

##### Heterogeneity between the Studies That Compared GDMtr vs. NGT

A high proportion of the observed heterogeneity in all the meta-analyses (as indicated by an I^2^ statistic > 90%) was due to underlying between-study differences [51]. We considered whether the GDM mothers achieved good glycaemic control with the treatments as one of the potential sources of heterogeneity. However, the information on the level of glycaemic control in GDM mothers was not reported in 40% of studies [27,30,31,32,33,38,40,43,45,46]. Therefore, the studies in which the authors stated that the mothers achieved good glycaemic control were separated from other studies, to see if the achievement of good glycaemic control mediated the relationship between GDM and infant adiposity. The test for subgroup differences indicated that there was no statistically significant subgroup effect of studies indicating GDM mothers achieving good glycaemic control on infant FM (*p =* 0.76), %FM (*p =* 0.15), triceps (*p =* 0.34) and subscapular SFT (*p =* 0.73).

The test for subgroup differences in ‘pre-2010′ vs. ‘post-2010′ studies showed a statistically significant subgroup effect on FM (*p =* 0.03, Figure S2) and %FM (*p =* 0.02, Figure S3). There was no significant difference in FM and %FM between IGDMtr and INGT in ‘post-2010′ studies, whereas, in ‘pre-2010′ studies, FM and %FM were significantly higher in IGDMtr compared to their counterparts. Further, subgroup analyses by infant body composition assessment technique were performed for infant FM and %FM. There was no significant effect (*p =* 0.28) of body composition technique on infant FM (Figure S4). Subgroup difference in %FM was significant (*p <* 0.00001); however, the number of studies and participants who contributed to subgroups were considerably different (Figure S5). %FM measured with ADP (0.93%, −1.61 to 3.47) or the Catalano et al. equation (1.93%, −0.56 to 4.43) did not significantly differ between IGDMtr and INGT. %FM measured by TOBEC (2.13%, 1.34 to 2.93) or the Dauncy et al. equation (4.90%, 4.06 to 5.74) was higher in IGDMtr. Leave-one-out sensitivity analysis demonstrated that removing the studies that had used the Catalano equation or TOBEC changed the overall effect for %FM to statistical non-significance. Of note, from the four studies that used ADP [21,22,40,44], three [21,22,44] affirmed good glycaemic control in mothers. Sensitivity analysis performed after removing the study [40] with no data on glycaemic control did not change the pooled result for the ADP subgroup. Moreover, leave-one-GDM-criteria-out sensitivity testing for FM and %FM did not show significant changes in the pooled effects. Specifically, the sensitivity analysis for White’s classification, which is different from other criteria that use an OGTT, did not significantly change the overall results for infant FM and %FM (Figure S6).

## 4. Discussion

We performed a systematic review and a meta-analysis of published studies (irrespective of the study designs) reporting adiposity in infants exposed to GDM controlled with therapeutic interventions. Treatments for GDM lowered newborn adiposity compared to no treatment, and there were no significant differences in adiposity in IGDMtr according to the mode of therapy; however, the evidence was insufficient due to the low number of available studies. IGDMtr had higher FM and %FM compared to INGT, but there was no significant difference in subcutaneous adiposity as measured by SFT.

Accelerated fat deposition in the foetus of GDM women can be reduced by strict glycaemic control [36]. Most women with GDM can control blood glucose with lifestyle changes such as diet modification, and increased physical activity; however, approximately one-third of women may require additional pharmacological treatments [14]. Oral diabetic medication is widely accepted by pregnant women in contrast to insulin because of easier storage, administration and lower cost [52], but unlike insulin, both metformin and glyburide cross the placenta [53]. Additionally, meta-analyses of risks and benefits of using insulin, metformin and glyburide in GDM women requiring drug treatment have shown that glyburide is inferior to both insulin and metformin, resulting in higher birth weights and increased risk of macrosomia, while metformin is associated with more preterm births than insulin [54]. On the other hand, insulin can bind to its specific receptor (in the placenta) to activate its signalling pathways; thus, insulin treatment still may have effects on placental and foetal growth [55]. One of the studies included in our review [7] reported that treatment with insulin in addition to lifestyle modification significantly increased the FM and %FM in IGDMtr as opposed to lifestyle intervention alone; however, the authors speculated that there might have been a confounding effect of other maternal factors associated with increased infant adiposity, as the former group of mothers (i.e., those who also received insulin) were characterised with higher pre-pregnancy weight and parity than their counterparts. Moreover, metformin and glyburide can impact foetal growth in opposite ways [56,57]. Glyburide controls maternal hyperglycaemia by stimulating insulin production. When glyburide is transported to the foetus through the placenta, it may also increase insulin secretion by the foetal pancreas that results in foetal overgrowth [56]. On the other hand, metformin inhibits glucose and amino acid transportation from the mother to the developing foetus through the placenta [57], which may cause foetal undergrowth. Despite this, the findings of the two RCTs included in our review [34,35] suggested that the effects of metformin, glyburide or insulin on infant adiposity were not significantly different; nonetheless, more studies are required for definitive conclusions. As reported in two recent systematic reviews, although there are no significant differences in body composition at birth [58], children exposed to metformin in utero show accelerated postnatal growth, compared to those exposed to insulin [59]. Therefore, tracking body composition trajectory of children exposed to pharmacological interventions in utero should be a research priority.

Our meta-analysis shows that treatments for GDM normalise newborns’ subcutaneous fat measured by SFT, but not overall adiposity measured by FM and %FM. These findings suggest that the phenotype of the IGDMtr may be distinguished with increased internal adiposity. Increased intra-abdominal adiposity is associated with several metabolic disorders, while superficial subcutaneous adiposity may exert a protective effect [60]. Furthermore, exposure to excess fuels in the gestational environment may lead to increased hepatic fat deposition in the foetus, which possibly plays a role in the development of nonalcoholic liver disease in children [61]. On the other hand, the accuracy of SFT measurements is dependent on the skills of the measurer, and the adiposity prediction equations with SFT are highly specific to the infant population that the data were derived from [62]. Thus, differentiating adipose tissue compartments with more reliable objective techniques and assessing hepatic fat deposition in IGDMtr and INGT at birth is important to identify these differences and any effects of GDM treatments. Comparing different adiposity compartments was beyond the scope of the current review, and such studies are very limited. Two small studies [40,63] reported that there were no significant differences in %FM, subcutaneous fat (cm^3^) and intra-abdominal fat/length (cm^2^) at 1–3 weeks [40], and in total adipose tissue (cm^3^), subcutaneous adipose tissue (cm^3^), internal abdominal adipose tissue (cm^3^) at 1–2 weeks [63] in IGDMtr and INGT infants. Intriguingly, one study reported a significant increase in intrahepatocellular lipid content in IGDMtr compared to INGT, while the other did not detect such a difference. However, glycaemic control was not described in the former study, whereas ~80% of mothers in the latter study had good glycaemic control with a mean third-trimester HbA1c level of 5.3%.

There were no significant differences in %FM in IGDMtr and INGT in studies ‘post-2010′ or when newborn overall adiposity was measured with ADP. These findings may be attributed to more intensive management of hyperglycaemia in the ‘post-2010′ period. Moreover, our findings highlight the importance of using more accurate and reliable objective infant body composition techniques such as ADP. The high degree of between-study heterogeneity may have arisen from the use of a wide variety of GDM diagnostic criteria, differences in the severity of hyperglycemia and level of glycaemic control, and confounding effects of maternal obesity, ethnicity, gestational weight gain, smoking, gestational age, infants’ sex and age at the investigation. Future studies should adopt universal criteria for the diagnosis of GDM, use reliable body composition assessment techniques such as ADP, and report the treatments and level of glycaemic control in GDM mothers throughout the pregnancy to enable robust conclusions on the association between GDM and newborn adiposity.

To our knowledge, the current review is the first to simultaneously evaluate studies reporting adiposity in newborns exposed to treated GDM vs. no treatment, different treatment regimens for GDM, and treated GDM vs. NGT. Adiposity in infants exposed to GDM compared to NGT has been investigated in a subgroup analysis of a previous systematic review [64] that examined the literature focused on the effect of all types of maternal diabetes. The authors found higher FM, %FM, triceps SFT and subscapular SFT in GDM-exposed infants compared to NGT; however, in some of the studies included in their meta-analysis (e.g., HAPO Study [11]), mothers were not treated. Other strengths of our study include the search of the literature in five major databases, investigation of differences in SFT sites such as abdominal and flank regions in addition to commonly reported triceps and subscapular measures, and investigation of potential sources of heterogeneity via subgroup and sensitivity analyses. Limitations of our study were that we only included studies published in English, excluded studies in which GDM status was self-reported by mothers where no information was reported on the use of treatments for glycaemic control, and considered only the most common measures of adiposity, i.e., FM, %FM and SFT, for comparison purposes.

## 5. Conclusions

Irrespective of the therapeutic strategy, treatment for GDM appears to reduce excess adiposity characteristic for newborns exposed to untreated GDM, but the evidence is limited. Due to the potential effects of oral hypoglycaemic medications on foetal growth, further studies on the impact of different GDM therapies on newborn adiposity are also warranted. Despite the significant heterogeneity found between the studies, our meta-analysis revealed higher overall adiposity (as measured with FM and %FM) but similar subcutaneous adiposity (as measured with SFT) in IGDMtr compared to INGT, suggesting that higher adiposity in IGDMtr may be due to excess fat accrual in internal adipose tissue. Future studies should distinguish adipose tissue distribution of IGDMtr and INGT with sufficient power to confirm these differences.

## Figures and Tables

**Table 1:** Characteristics and findings of the studies.

First Author, Year, Study Design, Time of Data Collection, Location	Study Groups *n* (Males%)	GDM Identification/Definition	Treatment(s)	Target Blood Glucose Levels (BGLs) and Level of Glycaemic Control	Infants’ Age	Infants’ Body Composition Assessment Method/SKINFOLD Thickness Measurements	Findings
(1) Treated GDM vs. no treatment for GDM							
**Landon, 2009** **Randomised trial** **2002–2007** **Bethesda, MD, USA** [36]	Control *=* 473 Treatment *=* 485	At 24th and 30th weeks using 4th International workshop conference criteria	Diet therapy (*n =* 427) and insulin (*n =* 36)	Targeted for fasting glucose <5.3 mmol/L or 2-h post-prandial glucose, <6.7 mmol/L Good glycaemic control achieved	Birth	FM was calculated as proposed by Catalano et al., 1995. Flank skinfold (data not given)	FM: Lower in treatment group (427 ± 198 vs. 464 ± 222, *p =* 0.003)
(2) Different treatment regimens for GDM							
*(a) Studies that measured only skinfolds*							
**Simmons, 1997** **1991–1992** **Middlemore Hospital and National Women’s Hospital, Auckland, New Zealand** [30]	All GDM Non-insulin *=* 11 (46%) Insulin *=* 9 (33%)	At 28–32 weeks gestation, using modified O’Sullivan criteria	All women received dietary therapy	Targeted fasting glucose >5.5 mmol/L and/or 2-h post-prandial glucose >7.0 mmol/L	<24 h	Subscapular	SFT: Not significantly different subscapular 5.4 (4.8–7.0) vs. 6.8 (5.0–7.9)
**Rowan, 2008** **randomised, open-label trial** **10 New Zealand and Australian urban ** **obstetrical hospitals** [34]	All GDM Metformin *=* 363 Insulin *=* 370	According to the criteria of the Australasian Diabetes in Pregnancy Society (ADIPS)	Metformin *=* 363 Insulin *=* 370	Aimed for the capillary glucose levels recommended by the ADIPS (after an overnight fast, <5.5 mmol/L; 2-h post-prandial level, <7.0 mmol/L	<48 h	Triceps and subscapular	SFT: Metformin group not significantly different from insulin group triceps (5.2 ± 1.6 vs. 5.1 ± 1.2, *p =* 0.30) subscapular (5.2 ± 1.5 vs. 5.2 ± 1.3, *p =* 0.60)
*(b) Studies that measured body composition*							
**Catalano, 2003** **Prospective cohort** **1990–2000** **Pregnancy Diabetes Clinic in Cleveland ** **Ohio, USA** [7]	NGT *=* 220 (54%) GDM *=* 195 (51%)	National Diabetes Data Group criteria	Diet only *=* 128 Diet + insulin *=* 67	Targeted fasting glucose >5.5 mmol/L and/or 2-h post-prandial glucose >6.7 mmol/L; Women maintained glucose values within the target range with diet and exercise (66%), plus insulin (34%)	<72 h	TOBEC	FM: Higher in diet + insulin group (492 ± 215 vs. 407 ± 196, *p =* 0.006) %FM: Higher in diet + insulin group (13.6 ± 4.6 vs. 11.7 ± 4.5, *p =* 0.007)
**Lain, 2009** **Randomised clinical trial** **2002–2005** **Magee-Women’s Hospital, Pittsburgh, Pennsylvania** [35]	Insulin *=* 41 (55.3%) Glyburide *=* 41 (58.5%)	Carpenter and Coustan criteria. Participants with a glucose level of >7.5 mmol/L had a 3-h 100-g OGTT	Insulin *=* 41 Glyburide *=* 41	Targeted fasting glucose >5.5 mmol/L and/or 2-h post-prandial glucose >6.7 mmol/L. Post-prandial dinner glucose was increased in the glyburide group.	<36 h	TOBEC Triceps, subscapular, suprailiac, and anterior thigh SFT (individual and sum given)	FM: Insulin group not significantly different from glyburide group (370 ± 167 vs. 473 ± 278, *p =* 0.06) %FM: Insulin group not significantly different from glyburide group (11.2 ± 4.2 vs. 12.8 ± 5.7, *p =* 0.18) SFT: Insulin group not significantly different from glyburide group triceps (3.9 ± 0.7 vs. 3.9 ± 0.9, *p =* 0.89), subscapular (4.1 ± 1.0 vs. 4.5 ± 1.3, *p =* 0.10), suprailiac (2.1 ± 0.6 vs. 2.1 ± 0.6, *p =* 0.85) and thigh (5.1 ± 1.2 vs. 5.4 ± 1.7, *p =* 0.28)
(3) Treated GDM vs. NGT				*^‡^ IGDMtr compared to INGT*			
*(a) Studies that measured only skinfolds*							
**Stevenson, 1991** **Cross-sectional** **USA** [28]	AGA NGT *=* 20 LGA NGT *=* 20 AGA GDM *=* 13	O’Sullivan and Mahan criteria	Dietary control	‘Well-managed GDM’	<72 h	Triceps	Not significantly different triceps compared to AGA NGT group (5.0 ± 1.1 vs. 4.3 ± 0.8, *p >* 0.05) and LGA NGT group (5.0 ± 1.1 vs. 6.2 ± 2.0, *p =* 0.058)
**Vohr, 1995** **Prospective longitudinal cohort** **1991–1993** **Women and Infants’ hospital, Rhode Island** [29]	AGA NGT *=* 69 AGA GDM = 62 LGA GDM = 57 LGA NGT = 74	Carpenter and Coustan criteria	Diet only *=* 385 Diet + insulin *=* 34 Diet includes 45–50% carbohydrates, 25% protein, and 25% fat.	Targeted fasting glucose >5.5 mmol/L and/or 2-h post-prandial glucose >6.7 mmol/L. The management team worked with all mothers to maintain BGL targets	20 ± 12 h	Triceps, subscapular, abdominal, suprailiac, and medial calf SFT	AGA GDM vs. AGA NGT Not significantly different triceps (3.5 ± 0.9 vs. 3.6 ± 0.8), subscapular (3.9 ± 1.0 vs. 3.9 ± 0.9), abdominal (3.5 ± 1.0 vs. 3.7 ± 0.9), suprailiac (3.4 ± 0.9 vs. 3.6 ± 1.0) and medial calf (4.8 ± 1.1 vs. 5.1 ± 1.1) LGA GDM vs. LGA NGT Not significantly different subscapular (5.5 ± 1.5 vs. 5.3 ± 1.3), suprailiac (4.9 ± 1.1 vs. 4.5 ± 1.1) and medial calf (6.7 ± 1.3 vs. 6.3 ± 1.1) significantly higher triceps (4.7 ± 1.0 vs. 4.5 ± 1.0) and abdominal (5.3 ± 1.4 vs. 4.9 ± 1.2) LGA GDM vs. AGA GDM Significantly higher subscapular (5.5 ± 1.5 vs. 3.9 ± 1.0), Abdominal (5.3 ± 1.4 vs. 3.5 ± 1.0), suprailiac (4.9 ± 1.1 vs. 3.4 ± 0.9) and medial calf (6.7 ± 1.3 vs. 4.8 ± 1.1) Not significantly different triceps (4.7 ± 1.0 vs. 3.5 ± 0.9)”
**Ng, 2004** **Cross-sectional** **Prince of Wales Hospital** **Hong Kong** [32]	NGT *=* 40 (50%) GDM *=* 42 (45.5%)	ADIPS criteria (1998)	Low-energy diet (1800 kcal/d)	Not reported	<24 h	Triceps and subscapular	SFT: Not significantly different triceps (4.8(4.2–5.1) vs. 4.7(4.1–5.5)) and subscapular (4.8(4.3–5.3) vs. 4.8(4.1–5.3), *p >* 0.05)
**Westage, 2006** **case-control** **1999–2001** **Middlemore Hospital,** **South Auckland** **New Zealand** [33]	NGT *=* 95 GDM *=* 138	Local criteria for diagnosis of GDM fasting glucose ≥5.5 mmol/Land/or a 2-h value after a 75 g glucose load ≥9.0 mmol/l	Insulin, usually as lispro insulin up to three times daily along with Humulin N if target fasting glucose exceeded two occasions or post-prandial readings were consistently high.	Target fasting glucose <5.5 mmol/L and post-prandial readings <6.5 mmol/l.	<24 h	Triceps and scapular	SFT: Significantly higher triceps (5.0 ± 1.2 vs. 4.4 ± 1.0) and scapular (5.6 ± 1.6 vs. 4.4 ± 1.0)
**Kara, 2017** **Cohort** **Ataturk University,** **Medical Hospital,** **Erzurum, Turkey** [42]	NGT *=* 20 GDM *=* 15groups were matched for gestational age and sex	At 24–28 gestational week using World Health Organisation (WHO) criteria	All were treated with dietary intervention, physical activity recommendation, and lifestyle management. All of them (diabetic) have used insulin therapy.	While the mean HbA1c level of mothers with gestational diabetes was 5.9 ± 1.7%, that of the controls was 5.2 ± 0.33%; there was no significant difference. Therefore, mothers with gestational diabetes were well controlled.	Birth	Triceps, Biceps, subscapular	SFT: Significantly higher triceps (3.9 ± 0.7 vs. 3.3 ± 1.1, *p =* 0.009) and subscapular (3.8 ± 0.8 vs. 3.4 ± 1.2, *p =* 0.04) Not significantly different biceps (2.8 ± 0.6 vs. 2.6 ± 0.9, *p =* 0.32)
**Mitanchez, 2017** **prospective cohort exposure-matched cohort** **2010–2013** **Paris, France** [43]	Lean NGT *=* 164 Lean GDM *=* 41 Obese NGT *=* 120 Obese GDM *=* 90	Fasting blood glucose (FBG) in the first trimester for women with BMI ≥30 kg/m^2^, and a 75 g OGTT between 24–28 weeks regardless of maternal BMI. Women were also screened for GDM at 32 weeks by performing a 75 g OGTT, regardless of maternal BMI. International Association of Diabetes and Pregnancy Study Groups (IADPSG) criteria	The first line treatment was dietary intervention with a standard 1800 kcal daily meal plan divided into three meals and snacks. Insulin treatment after two weeks of failed dietary therapy.	Target fasting glucose <5.0 mmol/L and post-prandial level <6.7 mmol/L.	<72 h	Triceps, biceps, suprailiac and subscapular	SFT: Normal weight group Not significantly different sum of SFT (triceps, biceps, subscapular, suprailiac) (18.6 ± 3.7 vs. 17.8 ± 3.1, *p >* 0.05) Obese group Not significantly different sum of SFT (19.9 ± 44 vs. 19.0 ± 3.5, *p >* 0.05)
**Prentice, 2019** **Prospective cohort** **2001–2009 and 2011–2013** **Rosie Maternity Hospital, Cambridge, UK** [46] **(additional data provided by authors)**	Earlier GDM *=* 98 (53%) Recent GDM *=* 122 (54%) Recent NGT *=* 876 (52%)	At around 28 weeks using IADPSG criteria	“Earlier” GDM was mostly treated with diet and lifestyle modification, with or without insulin. 19% of the ‘earlier’ GDM group were not diagnosed and did not receive any treatment. “Recent” all GDM women received standardised dietary and lifestyle advice and metformin and/or insulin if required.	Not reported	<8 days	Triceps, subscapular, flank, quadriceps	SFT: Earlier GDM Not significantly different sum of SFT (triceps, subscapular, flank, quadriceps) (26.0 ± 6.3 vs. 24.6 ± 6.0) Significantly higher skinfold SDS (0.31 ± 0.85 vs. 0.03 ± 0.86) Recent GDM Significantly lower sum of SFT (20.0 ± 3.6 vs. 24.6 ± 6.0) Significantly lower skinfold SDS (−0.41 ± 0.61 vs. 0.03 ± 0.86)
**Buhling, 2012** **Prospective cohort** **2005–2006 ** **Hamburg, Germany** [38] **(additional data provided by authors)**	NGT *=* 142 GDM *=* 30	GDM was defined according to the clinic’s guidelines, O’Sullivan criteria.	Treated with diet or diet + insulin.	Not reported	<72 h	Left anterior iliac spine, at the lower angle of the left scapula, at the middle of the femur, above the left quadriceps femoris and at the middle of the left triceps, midway between acromion and olecranon	SFT: Not significantly different all 4 sites triceps, 4.6 ± 0.9 vs. 4.8 ± 1.5, *p =* 0.67 scapular, 4.3 ± 1.41 vs. 4.1 ± 0.97, *p =* 0.54 iliac, 4.4 ± 1.3 vs. 4.2 ± 1.0, *p =* 0.45 femur, 5.2 ± 1.8 vs. 4.7 ± 1.4, *p =* 0.72
*(b) Studies that measured body composition*							
						‘Dauncy et al. equation [48]’	
**Enzi, 1980** **Cohort** **Italy** [27]	NGT *=* 17 GDM *=* 17	White’s classification, class A (abnormal glucose tolerance that reverted to normal postpartum)	Low-carbohydrate diet	Not reported	Birth	FM and FM% calculated by Dauncy et al. equation Sum of subscapular, subcostal, tricipital, and crural SFT	FM: Not significantly different (553 ± 49 vs. 386 ± 22) %FM: Significantly higher (17.1 ± 1.7 vs. 12.2 ± 0.5) SFT: Significantly higher sum of SFT (23.0 ± 1.4 vs. 17.8 ± 0.7)
**Naf, 2012Prospective case-control ** **Joan XXIII University Hospital, Tarragona, Spain** [39]	NGT *=* 130 (46.1%) GDM *=* 84 (53.2%)	National Diabetes Data Group criteria were used to define GDM before 30 weeks	Diet *=* 48 Diet + insulin *=* 29	Target fasting glucose values <5.3 mmol/L and or 1-h post-prandial values <7.8 mmol/L. GDM women had higher levels of fasting glucose4.5 ± 0.4 vs. 4.8 ± 0.6 mmol/L	<48 h	FM by Dauncy et al. equation. Triceps, biceps, subscapular, and flank skinfold thickness (data not given)	FM: Not significantly different (291 ± 131 vs. 318 ± 133, *p =* 0.198)
						‘Weststrate and Deurenberg equation [49]’	
**Ubel, 2014** **Cohort** **Abteilung für Geburtshilfe und Perinatalmedizin** **der Frauenklinik, Klinikum rechts der Isar, Technische** **Universität München** **Munich, Germany** [41]	Lean NGT *=* 15 (46.7%) Obese NGT *=* 13 (61.5%) Obese GDM *=* 16 (81.3%)	Hyperglycaemia and Pregnancy Outcome (HAPO) criteria	Diet *=* 7 Insulin treated *=* 9	fasting BGL at 3rd trimester did not significantly differ between the groups and was <5.1 mmol/L	1 week	FM by the equations of Weststrate and Deurenberg Sum of Biceps, triceps, subscapular, suprailiac	FM: Significantly higher compared to lean NGT (694 ± 117, vs. 583 ± 139, *p <* 0.05); Not significantly different compared to obese NGT (694 ± 117, vs. 660 ± 114, *p >* 0.05) SFT: Significantly higher compared to lean NGT (21.6 ± 2.4 vs. 18.9 ± 3.1) Not significantly different compared to obese NGT (21.6 ± 2.4 vs. 20.3 ± 2.6)
						‘Catalano et al. equation [50]’	
**Aman, 2011** **Case-control** **Örebro University Hospital, Sweden** [20]	NGT = 28 GDM = 10	2-h capillary whole-blood glucose concentration above 11 mmol/L, following a 75 g OGTT after 24th week of pregnancy	Dietary adjustments and multiple pre-meal insulin injections.	Daily blood glucose target, HbA1c 3.5–5.3% Glycaemic control was fairly good, with mean HbA1c values below the upper reference limit for healthy from the 24th to the 36th week of gestation.	<2 days	FM by Catalano et al., equation. Triceps, subscapular and abdomen flank SFT	FM: Significantly higher (700 ± 200 vs. 500 ± 200, *p <* 0.01) %FM: Significantly higher (17.0 ± 3.2 vs. 13.5 ± 3.5, *p <* 0.01) SFT: Significantly higher in triceps (6.6 ± 1.7 vs. 5.3 ± 1.1, *p <* 0.05) and subscapular (6.0 ± 2.1 vs. 4.8 ± 1.1, *p <* 0.05)Not significantly different in abdominal flank (5.1 ± 1.5 vs. 3.9 ± 1.0, *p >* 0.05)
**Schaefer-Graf,2011** **Cohort** **2007–2008** **Vivantes Medical Centre, Berlin, Germany** [37]	NGT *=* 190 (48.4%) GDM *=* 150 (44.0%)	American Diabetes Association criteria for measurements in venous plasma. With respect to lower glucose concentrations in capillary compared with venous blood, the threshold for fasting glucose was modified into 5.0 mmol/L, while post challenge capillary glucose levels correspond with those in venous blood.	Dietary instruction and performed self-monitoring of BGL. Insulin therapy given before 36 weeks gestation based on BGL and/or foetal abdominal circumference (AC).	fasting <5.0 mmol/L or 2-h postprandial <6.7 mmol/L or when AC > 75th percentile fasting <5.0 mmol/L or 2-h postprandial <11.1 mmol/L ‘Well-controlled’ Maternal serum glucose levels did not differ between control subjects and women with GDM	<48 h	FM by Catalano et al., equation.	FM: Significantly higher (433 ± 14 vs. 381 ± 13, *p <* 0.01)
**Maple-Brown, 2019** **Longitudinal cohort study** **2011–2017** **Northern Territory, Australia** [45]	Indigenous NGT *=* 117 Indigenous GDM/DI *p* = 278 Non-indigenous NGT *=* 118 Non-indigenous GDM/DIP* *=* 461	GDM were diagnosed by either the ADIPS guidelines or a universal 75 gm OGTT and revised glucose cut points as recommended by the WHO. DIP, was defined as diabetes first identified in pregnancy, but with glucose or HbA1c values higher glucose than GDM), and identified from medical records	Diet only or Metformin only or Insulin only or Metformin and insulin	Not reported	<72 h	FM by Catalano et al. equation.	FM: Not significantly different (11.3 ± 4.2 vs. 11.5 ± 3.7, *p =* 0.65) Non-indigenous Significantly lower (10.2 ± 3.7 vs. 11.5 ± 3.5, *p =* 0.0006)
**Samsuddin, 2020** **Prospective cohort** **2014–2017** **Tertiary antenatal clinic, Kuala Lumpur, Malaysia** [47]	Obese NGT *=* 94 Non-obese NGT *=* 268 GDM *=* 145 BMI categories (Asian) Normal:18.5–22.9 kg/m^2^; Overweight: 23–27.4 kg/m^2^; Obese: ≥27.5 kg/m^2^	FPG ≥ 5.1 mmol/L and/or 2-h glucose ≥7.8 mmol/L after a 75 g OGTT (based on the study centre’s definition and the Malaysian 2015 Clinical Practice Guideline	Nutrition therapy. If >30% of the self-monitoring of blood glucose values is beyond target despite compliance with medical nutrition therapy, insulin therapy is initiated	The glycaemic targets for GDM in the study centre: fasting 3.5–5.1 mmol/L, pre-meals 4.0–5.8 mmol/L, 2-h post-prandial 4.0–6.7 mmol/L. Well-treated GDM mothers (pre-delivery HbA1c 5.3%)	<24 h	FM by Catalano et al. equation. Sum of flank, triceps, subscapular SFT	FM: Not significantly different compared to non-obese NGT (909 ± 113 vs. 924 ± 149, *p >* 0.05)Significantly lower compared to obese NGT (909 ± 113 vs. 973 ± 149, *p <* 0.05) SFT: Significantly lower sum of SFT (flank, triceps, subscapular) compared to obese NGT (14.2 ± 3.0 vs. 16.1 ± 5.3, *p <* 0.05) Not significantly different compared to non-obese NGT (14.2 ± 3.0 vs. 14.4 ± 2.8, *p >* 0.05)
						‘TOBEC’	
**Okereke, 2001** **Cohort** **1998–2000** **Metro Health Medical** **Centre, Cleveland, USA** [31]	NGT *=* 44 (58.8%) GDM *=* 34 (59.1%)	Carpenter and Coustan criteria	Diet *=* 23 Diet + insulin *=* 11	Not reported	<48 h	TOBEC paediatric model HP-2	FM: Significantly higher (480 ± 210 vs. 360 ± 150, *p =* 0.01) %FM: Significantly higher (13.2 ± 4.3 vs. 10.5 ± 3.8, *p =* 0.01)
**Catalano, 2003** **Prospective cohort** **1990–2000Pregnancy Diabetes Clinic in Cleveland ** **Ohio, USA** [7]	NGT *=* 220 (54%) GDM *=* 195 (51%)	At 26 to 28 weeks using National Diabetes Data Group criteria	Diet only *=* 128 Diet + insulin *=* 67	Targeted fasting glucose >5.5 mmol/L and/or 2-h post-prandial glucose >6.7 mmol/L. Women maintained glucose values within the target range with diet and exercise (66%), plus insulin (34%).	<72 h	TOBEC Triceps and subscapular, flank, thigh, abdominal SFT	FM: Significantly higher (436 ± 206 vs. 362 ± 198, *p =* 0.0002) %FM: Significantly higher (12.4 ± 4.6 vs. 10.4 ± 4.6, *p =* 0.0001) SFT: Significantly higher at all 5 sites triceps (4.7 ± 1.1 vs. 4.2 ± 1.3, p *=* 0.0001) subscapular (5.4 ± 1.4 vs. 4.6 ± 1.2, *p =* 0.0001) flank (4.2 ± 1.2 vs. 3.8 ± 1.0, *p =* 0.0001) thigh (6.0 ± 1.4 vs. 5.4 ± 1.5, *p =* 0.0001) abdominal wall (3.5 ± 0.9 vs. 3.0 ± 0.8, *p =* 0.0001)
						‘ADP (Pea Pod)’	
**Brumbaugh, 2013** **Cross-sectional** **University of Colorado Hospital or Denver Health.** **Colorado, USA** [40]	Normal NGT *=* 13 (53.8%) Obese/GDM *=* 12 (66.7%) Both groups matched for ethnicity	At 24–28 weeks using Carpenter and Coustan criteria	2 were diet control, 10 were requiring insulin or glyburide.	Not reported	1–3 weeks	ADP (Pea Pod) Sum of triceps and subscapular SFT	%FM: Not significantly different 14.7 ± 3.0 vs. 13.1 ± 5.0, *p =* 0.36 SFT:Significantly higher sum of SFT (11.7 ± 1.3 vs. 9.9 ± 2.0, *p =* 0.01
**Lingwood, 2011** **Prospective cohort** **2009–2010^a^** **Royal Brisbane and Women’s Hospital** **Queensland, Australia** [21] **(additional data provided by authors)**	NGT *=* 77 (53%) GDM *=* 84 (50%)	ADIPS criteria	Dietary and physical activity advice. Insulin treatment was begun if more than two glucose measurements exceeded the target range in 1 week.	Target BGLs were set according to current ADIPS guidelines: 5.5 mmol/L or lower fasting, and 7.0 mmol/L or lower 2-h post-prandial. 80% met both current fasting and post-prandial ADIPS targets. 75% met the lower targets of the American Diabetes Association (5.3 and 6.7 mmol/L)	<6 days	ADP (Pea Pod)	FM: Significantly higher (413 ± 192 vs. 350 ± 162, *p =* 0.003) %FM: Significantly higher (12.1 ± 4.3 vs. 10.1 ± 4.1, *p =* 0.003)
**Au, 2013** **Cross-sectional** **September-October 2010** **Royal Prince Alfred Hospital** **Sydney, Australia** [22]	NGT *=* 532 (53%) GDM *=* 67 (42%)	ADIPS criteria.	Dietary and physical activity advice. Insulin therapy was commenced when glycaemic targets could not be met.	Good glycaemic control was achieved in 90% of women meeting both fasting and post-prandial ADIPS targets	<48 h	ADP (Pea Pod)	%FM: Not significantly different 7.9 ± 4.5 vs. 9.3 ± 4.3, *p =* 0.018
**Andersson-Hall, 2018** **Longitudinal cohort** **2009–2018** **6 antenatal health units and Sahlgrenska University Hospital** **Gothenburg, Sweden** [44]	Normal weight group 83 (50.6%) Obese group 26 (65.4%) GDM group 26 (38.5%)	All pregnant women had non-fasting blood glucose measured regularly throughout pregnancy, and women with an elevated non-fasting glucose (>8 mmol/L) underwent OGTT. GDM mothers were identified based on the European Association for the Study of Diabetes criteria, at 27 ± 7 gestational weeks.	All 26 received diet and lifestyle advice, 4 received insulin.	Not reported	4–10 days	ADP (Pea Pod)	FM: Normal weight group Significantly different (640 ± 200 vs. 500 ± 230, *p =* 0.0034) Obese group Not significantly different sum of SFT (640 ± 200 vs. 580 ± 170, *p =* 0.29) %FM: Normal weight group Significantly different (16.44 ± 4.68 vs. 13.5 ± 4.6, *p =* 0.0036) Obese group Not significantly different sum of SFT (16.44 ± 4.68 vs. 15.23 ± 3.86, *p =* 0.26)

Studies are grouped according to the type of the outcome, and within these groups, the studies are subgrouped according to the body composition technique used. %FM: percent fat mass; ADP: air displacement plethysmography; AGA: appropriate for gestational age; BMI: body mass index (kg/m^2^); FM: fat mass (g); GDM: gestational diabetes mellitus; IGDMtr: infants exposed to treated GDM; INGT: infants exposed to NGT; LGA: large for gestational age; NGT: normal glucose tolerance; OGTT: oral glucose tolerance test; SFT: skinfold thickness (mm); TOBEC: total body electrical conductivity; *DIP: diabetes in pregnancy (defined as diabetes first identified in pregnancy, but meeting glucose or HbA1c values diagnostic of overt diabetes outside pregnancy); ***^‡^*** body composition data for ‘treated GDM vs. NGT’ subgroup are presented as IGDMtr vs. INGT.3.2.1. GDM screening criteria and target blood glucose concentrations.

**Table 2:** Quality assessment of the studies included in the review, using the Evidence Project risk of bias tool.

First Author	Year	Evidence Project Risk of Bias Tool Items									
		(1) Cohort	(2) Control or Comparison Group	(3) Pre/Post Intervention Data	(4) Random Assignment of Participants to the Intervention	(5) Random Selection of Participants for Assessment	(6) Follow-Up Rate of 80% or More ^a^		(7) Comparison Groups Equivalent on Sociodemographic ^b^		(8) Comparison Groups Equivalent at Baseline on Disclosure
		Judgement	Judgement	Judgement	Judgement	Judgement	Judgement	Follow-U p Rate	Judgement	Comment	Judgement
**Enzi**	1980	Yes	Yes	No	NA	No	Yes	87.5%	NR		NA
**Stevenson**	1991	No	Yes	No	NA	No	NA		NR		NA
**Vohr**	1995	Yes	Yes	No	NA	No	Yes	100%	NR		NA
**Simmons**	1997	Yes	Yes	No	NA	No	No	57%	Yes	Ethnicity and sex not significantly different	NA
**Okereke**	2001	Yes	Yes	No	NA	No	Yes	100%	Partial	Sex not significantly different, ethnicity significantly different	NA
**Catalano**	2003	Yes	Yes	No	NA	No	Yes	100%	Partial	Ethnicity significantly different, sex not significantly different	NA
**Ng**	2004	No	Yes	No	NA	No	NA		Partial	Sex not significantly different	NA
**Westgate**	2006	No	Yes	No	NA	No	NA		Yes	Sex and ethnicity not significantly different	NA
**Rowan**	2008	Yes	Yes	No	Yes	No	Yes	97.6%	Partial	Ethnicity not significantly different	NA
**Lain**	2009	Yes	Yes	No	Yes	No	Yes	82.8%	Yes		NA
**Landon**	2009	Yes	Yes	No	Yes	No	Yes	93.9%	Partial	Ethnicity not significantly different	NA
**Aman**	2011	No	Yes	No	NA	No	NA		NR		NA
**Lingwood**	2011	Yes	Yes	No	NA	No	Yes	100%	NR		NA
**Naf**	2011	Yes	Yes	No	NA	No	Yes	100%	Partial	Sex not significantly different	NA
**Schaefer-Graf**	2011	Yes	Yes	No	NA	No	Yes	100%	Partial	Sex not significantly different	NA
**Au**	2012	No	Yes	No	NA	No	NA		No	Significant difference in maternal ethnicity	NA
**Buhling**	2012	Yes	Yes	No	NA	No	Yes	100%	Partial	Ethnicity not significantly different	NA
**Brumbaugh**	2013	No	Yes	No	NA	No	NA		Yes	Sex and ethnicity not significantly different	NA
**Ubel**	2014	Yes	Yes	No	NA	No	Yes	100%	No	Sex significantly different	NA
**Mitanchez**	2017	yes	Yes	No	NA	No	Yes	90.3%	NR		NA
**Kara**	2017	Yes	Yes	No	NA	No	Yes	100%	Partial	Sex not significantly different	NA
**Andersson-Hall**	2018	Yes	Yes	No	NA	No	Yes	83%	Partial	Sex not significantly different	NA
**Maple-Brown**	2019	Yes	Yes	No	NA	No	Yes	100%	NR		NA
**Prentice**	2019	Yes	Yes	No	NA	No	NR		Partial	Sex not significantly different	NA
**Samsuddin**	2020	Yes	Yes	No	NA	No	Yes	100%	No	Ethnicity significantly different	NA

NA: not applicable; NR: not reported. Studies are ordered according to the year of the publication. ^a^ Follow-up rate was calculated as the number of participants at the final assessment*100 divided by the number of participants at the first assessment, as stated in the paper. ^b^ Infant sex and ethnicity were considered as sociodemographic characteristics. If the authors have only reported that the study arms are equivalent on one of the sociodemographic characteristics, it was indicated as “Partial”, while if the study arms were not equivalent at least on one of the socio-demographics, it was decided that the criterion was not met (“No”).

## Data Availability

Data sharing not applicable.

## Supplementary Materials

The following are available online at https://www.mdpi.com/2077-0383/10/4/835/s1. Table S1: Diagnostic Criteria for gestational diabetes mellitus (GDM) used by the studies included in the review; Figure S1: Search strategy in Medline via Ovid; Figure S2: Forest plot comparing fat mass (g) in infants exposed to treated gestational diabetes mellitus (IGDM) and infants exposed to normal glucose tolerance (INGT) by subgroup analysis of time of the study; ‘pre-2010′ vs. ‘post-2010′; Figure S3: Forest plot comparing percent fat mass (%) in infants exposed to treated gestational diabetes mellitus (IGDM) and infants exposed to normal glucose tolerance (INGT) by subgroup analysis of time of the study; ‘pre-2010′ vs. ‘post-2010′; Figure S4: Forest plot comparing fat mass (g) in infants exposed to treated gestational diabetes mellitus (IGDM) and infants exposed to normal glucose tolerance (INGT) by subgroup analysis of infant body composition assessment technique; Figure S5: Forest plot comparing percent fat mass (%) in infants exposed to treated gestational diabetes mellitus (IGDM) and infants exposed to normal glucose tolerance (INGT) by subgroup analysis of infant body composition assessment technique. Figure S6: Forest plot comparing percent fat mass (a) and percent fat mass (b) in infants exposed to treated gestational diabetes mellitus (IGDM) and infants exposed to normal glucose tolerance (INGT) excluding the effect by the study used White’s classification for diagnosis of gestational diabetes.
