# Supplementary material for: Gestational Diabetes Mellitus and Infant Adiposity at Birth: A Systematic Review and Meta-Analysis of Therapeutic Interventions
Source: J Clin Med. 2021 Feb 18;10(4):835. doi: 10.3390/jcm10040835 (PMC7922793; doi:10.3390/jcm10040835)
Supplement: Supplementary file 1 [file jcm-10-00835-s001.pdf]

Table S1. Diagnostic Criteria for gestational diabetes mellitus (GDM) used by the studies included in the review.

Criteria name	Year	Screening oral glucose challenge test (OGCT)		Diagnostic oral glucose tolerance test (OGTT)						Studies in the review
		Dose	1 h mmol/L	Fasting mmol/L	Dose	1 h mmol/L	2 h mmol/L	3 h mmol/L	Abnormal values	
O' Sullivan and Mahan [51]	1964	50 g fasting	140	5.0	100 g	9.2	8.1	6.9	2 or more	[18]
White's Classification [52]	1978	Pregnant women are categorised according to duration and age of onset of diabetes								[17]
US National Diabetes Data Group [53]	1979	50 g fasting	7.8	5.8	100 g	10.5	9.1	8.0	2 or more	[6,29]
Carpenter and Coustan [54]	1982	50 g fasting	7.2	5.3	100 g	10.0	8.6	7.8	2 or more	[19,21,25,30]
Modified O'Sullivan Criteria by Court et al. [55]	1985	50 g fasting	7.8	5.8	100 g	10.0	8.9	7.8	2 or more	[20]
European Association for the Study of Diabetes (EASD) [56]	1991	_	_	7.0	75 g	11.0	9.0	_	Either the 0-h or 1-h concentration should meet or exceed stated values in addition to a 2-h value	[34]
Australasian Diabetes In Pregnancy Society (ADIPS) [57]	1998	50 g or 75 g non-fasting	50 g: 7.8 or 75 g: 8.0	5.5	75 g	_	8.0 or 9.0 *	_	1 or more	[11,12,22,24]
4th International workshop conference [58]	1998	50 g fasting	7.2 or 7.8	5.3	100 g or 75 g	10.0	8.6	7.8 (3 hr value only for 100 g test)	2 or more	[26]
American Diabetes Association (ADA) [59]	2004	50 g fasting	7.2 or 7.8	5.3	100 g or 75 g	10.0	8.6	7.8 (3 hr value only for 100 g test)	2 or more	[27,28]
International Association of Diabetes and Pregnancy Study Groups (IADPSG) [60]	2010	_	_	5.1	75 g	10.0	8.5	_	1 or more	[31,33,35,36]
World Health Organisation [61]	2014	_	_	5.1–6.9	75 g	10.0	8.5–11.0	_	1 or more	[32]

Guidelines are ordered according to the year of the publication. All values are for venous plasma glucose, except for O'Sullivan and Mahan criteria, which are venous whole blood glucose. IADPSG criteria are based on the Hyperglycaemia and Pregnancy Outcome (HAPO) study. OGCT: oral glucose challenge test; OGTT: oral glucose tolerance test.

Step	Searches
**1**	(gestation* or pregnan* or maternal or mother*).mp. [mp=title, abstract, original title, name of substance word, subject heading word, floating sub-heading word, keyword heading word, organism supplementary concept word, protocol supplementary concept word, rare disease supplementary concept word, unique identifier, synonyms]
**2**	(diabet* or hyperglyc?mi* or glyc?mi* or glucose intolerance or impaired glucose tolerance or insulin resistance).mp. [mp=title, abstract, original title, name of substance word, subject heading word, floating sub-heading word, keyword heading word, organism supplementary concept word, protocol supplementary concept word, rare disease supplementary concept word, unique identifier, synonyms]
**3**	(infan* or neonat* or child* or newborn* or offspring* or birth).mp. [mp=title, abstract, original title, name of substance word, subject heading word, floating sub-heading word, keyword heading word, organism supplementary concept word, protocol supplementary concept word, rare disease supplementary concept word, unique identifier, synonyms]
**4**	(“body composition” or fat or adiposity).mp. [mp=title, abstract, original title, name of substance word, subject heading word, floating sub-heading word, keyword heading word, organism supplementary concept word, protocol supplementary concept word, rare disease supplementary concept word, unique identifier, synonyms]
**5**	("air-displacement plethysmography" or "isotope dilution" or "deuterium dilution" or "bioelectrical impedance" or "total body electrical conductivity" or "dual-energy x-ray absorptiometry" or "photon-counting computed tomography" or "photon absorptiometry" or "total body potassium" or "magnetic resonance imaging" or "skinfold" or "skin fold" or anthropometry).mp. [mp=title, abstract, original title, name of substance word, subject heading word, floating sub-heading word, keyword heading word, organism supplementary concept word, protocol supplementary concept word, rare disease supplementary concept word, unique identifier, synonyms]
**6**	1 and 2 and 3 and 4 and 5
**7**	limit 6 to (english language and humans and “all infant (birth to 23 months)”)

Figure S1. Search strategy in Medline via Ovid.

Study or Subgroup	IGDMtr Mean [g]	IGDMtr SD [g]	IGDMtr Total	INGT Mean [g]	INGT SD [g]	INGT Total	Weight	Mean Difference IV, Random, 95% CI	Year
**1.2.1 "Pre-2010"**									
Enzi 1980 [27]	539	49	17	386	22	17	12.3%	153.00 [127.47, 178.53]	1980
Okereke 2002 [31]	480	210	34	360	150	44	8.1%	120.00 [36.65, 203.35]	2002
Catalano 2003 [7]	436	206	195	362	198	220	11.4%	74.00 [35.01, 112.99]	2003
Lingwood 2011 [21]	413	192	84	350	162	77	10.3%	63.00 [8.27, 117.73]	2011
Aman 2011 [20]	700	200	10	500	200	28	4.6%	200.00 [55.59, 344.41]	2011
Schaefer-Graf 2011 [37]	433	14	150	381	13	190	12.9%	52.00 [49.10, 54.90]	2011
**Subtotal (95% CI)**			**490**			**576**	**59.6%**	**99.57 [49.44, 149.71]**	
Heterogeneity: Tau² = 3013.81; Chi² = 66.94, df = 5 (P < 0.00001); I² = 93%									
Test for overall effect: Z = 3.89 (P < 0.0001)									
**1.2.2 "Post-2010"**									
Naf 2012 [39]	291	131	84	318	133	130	11.6%	-27.00 [-63.16, 9.16]	2012
Ubel 2014 [41]	694	117	16	619	132	28	8.7%	75.00 [-0.35, 150.35]	2014
Anderson 2019 [44]	640	200	26	520	230	109	7.7%	120.00 [31.83, 208.17]	2019
Samsudin 2020 [47]	909	113	145	937	131	362	12.4%	-28.00 [-50.81, -5.19]	2020
**Subtotal (95% CI)**			**271**			**629**	**40.4%**	**19.76 [-34.13, 73.64]**	
Heterogeneity: Tau² = 2216.26; Chi² = 16.16, df = 3 (P = 0.001); I² = 81%									
Test for overall effect: Z = 0.72 (P = 0.47)									
**Total (95% CI)**			**761**			**1205**	**100.0%**	**68.46 [29.91, 107.01]**	
Heterogeneity: Tau² = 2986.33; Chi² = 136.30, df = 9 (P < 0.00001); I² = 93%									
Test for overall effect: Z = 3.48 (P = 0.0005)									
Test for subgroup differences: Chi² = 4.52, df = 1 (P = 0.03), I² = 77.9%									

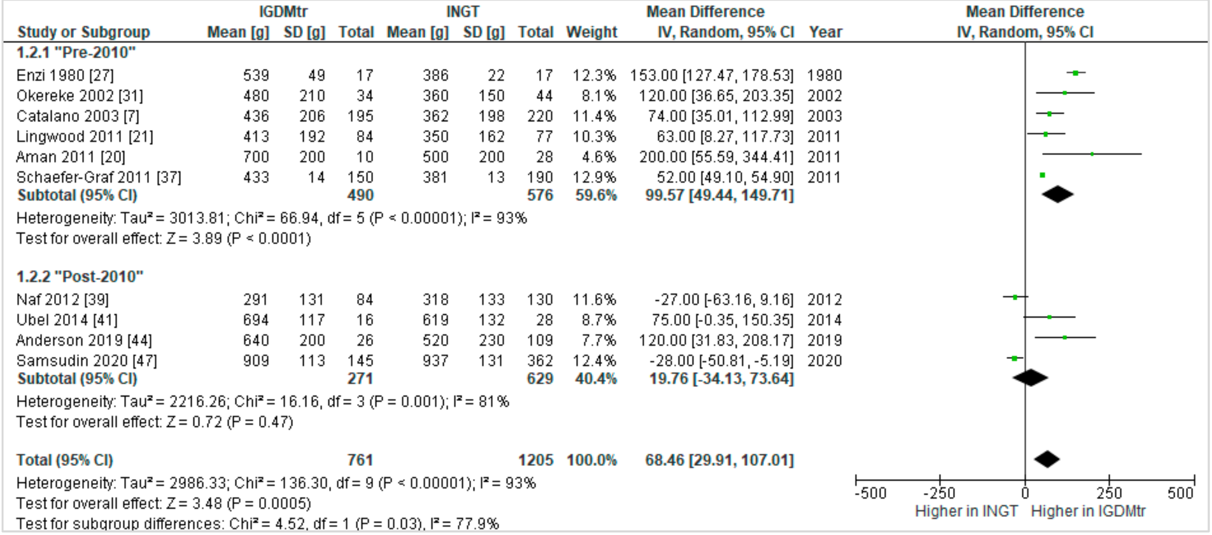

Figure S2. Forest plot comparing fat mass (g) in infants exposed to treated gestational diabetes mellitus (IGDMtr) and infants exposed to normal glucose tolerance (INGT) by subgroup analysis of time of the study; 'pre-2010' vs. 'post-2010'.

Study or Subgroup	IGDMtr Mean	IGDMtr SD	IGDMtr Total	INGT Mean	INGT SD	INGT Total	Weight	Std. Mean Difference IV, Random, 95% CI
**1.3.1 "Pre-2010"**								
Aman 2011 [20]	17	3.2	10	13.5	3.5	28	8.6%	1.00 [0.24, 1.76]
Catalano 2003 [7]	12.4	4.6	195	10.4	4.6	220	13.8%	0.43 [0.24, 0.63]
Enzi 1980 [27]	17.1	1.7	17	12.2	0.5	17	5.5%	3.82 [2.64, 4.99]
Lingwood 2011 [21]	12.1	4.3	84	10.1	4.1	77	12.9%	0.47 [0.16, 0.79]
Okereke 2002 [31]	13.2	4.3	34	10.5	3.8	44	11.6%	0.66 [0.20, 1.12]
**Subtotal (95% CI)**			**340**			**386**	**52.4%**	**0.99 [0.44, 1.54]**
Heterogeneity: Tau² = 0.30; Chi² = 32.83, df = 4 (P < 0.00001); I² = 88%								
Test for overall effect: Z = 3.54 (P = 0.0004)								
**1.3.2 "Post-2010"**								
Anderson 2019 [44]	16.4	4.7	26	13.9	4.5	109	11.8%	0.55 [0.11, 0.98]
Au 2013 [22]	7.3	4.5	67	9.3	4.3	532	13.4%	-0.46 [-0.72, -0.21]
Brumbaugh 2013 [40]	14.7	3	12	13.1	5	13	8.3%	0.37 [-0.42, 1.16]
Maple-Brown 2019 [45]	11.5	3.6	235	10.6	3.9	739	14.1%	0.23 [0.09, 0.38]
**Subtotal (95% CI)**			**340**			**1393**	**47.6%**	**0.14 [-0.33, 0.60]**
Heterogeneity: Tau² = 0.18; Chi² = 26.38, df = 3 (P < 0.00001); I² = 89%								
Test for overall effect: Z = 0.57 (P = 0.57)								
**Total (95% CI)**			**680**			**1779**	**100.0%**	**0.56 [0.21, 0.91]**
Heterogeneity: Tau² = 0.22; Chi² = 79.04, df = 8 (P < 0.00001); I² = 90%								
Test for overall effect: Z = 3.13 (P = 0.002)								
Test for subgroup differences: Chi² = 5.42, df = 1 (P = 0.02), I² = 81.6%								

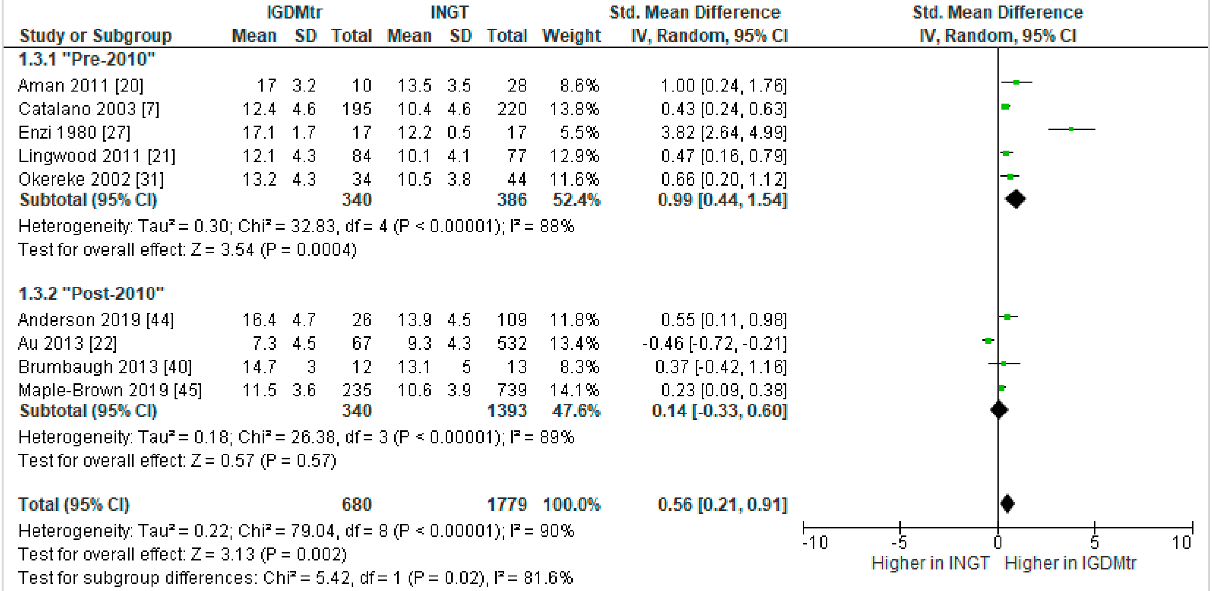

Figure S3. Forest plot comparing percent fat mass (%) in infants exposed to treated gestational diabetes mellitus (IGDMtr) and infants exposed to normal glucose tolerance (INGT) by subgroup analysis of time of the study; 'pre-2010' vs. 'post-2010'.

Study or Subgroup	IGDMtr Mean [g]	IGDMtr SD [g]	IGDMtr Total	INGT Mean [g]	INGT SD [g]	INGT Total	Weight	Mean Difference IV, Random, 95% CI	Year
**1.4.1 Catalano equation**									
Aman 2011 [20]	700	200	10	500	200	28	4.6%	200.00 [55.59, 344.41]	2011
Schaefer-Graf 2011 [37]	433	14	150	381	13	190	12.9%	52.00 [49.10, 54.90]	2011
Samsudin 2020 [47]	909	113	145	937	131	362	12.4%	-28.00 [-50.81, -5.19]	2020
**Subtotal (95% CI)**			**305**			**580**	**29.9%**	**42.90 [-30.37, 116.17]**	
Heterogeneity: Tau² = 3263.34; Chi² = 50.59, df = 2 (P < 0.00001); I² = 96%									
Test for overall effect: Z = 1.15 (P = 0.25)									
**1.4.2 TOBEC**									
Okereke 2002 [31]	480	210	34	360	150	44	8.1%	120.00 [36.65, 203.35]	2002
Catalano 2003 [7]	436	206	195	362	198	220	11.4%	74.00 [35.01, 112.99]	2003
**Subtotal (95% CI)**			**229**			**264**	**19.5%**	**82.26 [46.94, 117.58]**	
Heterogeneity: Tau² = 0.00; Chi² = 0.96, df = 1 (P = 0.33); I² = 0%									
Test for overall effect: Z = 4.56 (P < 0.00001)									
**1.4.3 Dauncy equation**									
Enzi 1980 [27]	539	49	17	386	22	17	12.3%	153.00 [127.47, 178.53]	1980
Naf 2012 [39]	291	131	84	318	133	130	11.6%	-27.00 [-63.16, 9.16]	2012
**Subtotal (95% CI)**			**101**			**147**	**23.9%**	**63.47 [-112.92, 239.87]**	
Heterogeneity: Tau² = 15944.96; Chi² = 63.52, df = 1 (P < 0.00001); I² = 98%									
Test for overall effect: Z = 0.71 (P = 0.48)									
**1.4.4 ADP (Pea Pod)**									
Lingwood 2011 [21]	413	192	84	350	162	77	10.3%	63.00 [8.27, 117.73]	2011
Anderson 2019 [44]	640	200	26	520	230	109	7.7%	120.00 [31.83, 208.17]	2019
**Subtotal (95% CI)**			**110**			**186**	**18.0%**	**80.59 [28.99, 132.19]**	
Heterogeneity: Tau² = 222.76; Chi² = 1.16, df = 1 (P = 0.28); I² = 14%									
Test for overall effect: Z = 3.06 (P = 0.002)									
**1.4.5 Weststrate and Deurenberg equation**									
Ubel 2014 [41]	694	117	16	619	132	28	8.7%	75.00 [-0.35, 150.35]	2014
**Subtotal (95% CI)**			**16**			**28**	**8.7%**	**75.00 [-0.35, 150.35]**	
Heterogeneity: Not applicable									
Test for overall effect: Z = 1.95 (P = 0.05)									
**Total (95% CI)**			**761**			**1205**	**100.0%**	**68.46 [29.91, 107.01]**	
Heterogeneity: Tau² = 2986.33; Chi² = 136.30, df = 9 (P < 0.00001); I² = 93%									
Test for overall effect: Z = 3.48 (P = 0.0005)									
Test for subgroup differences: Chi² = 0.95, df = 4 (P = 0.92), I² = 0%									

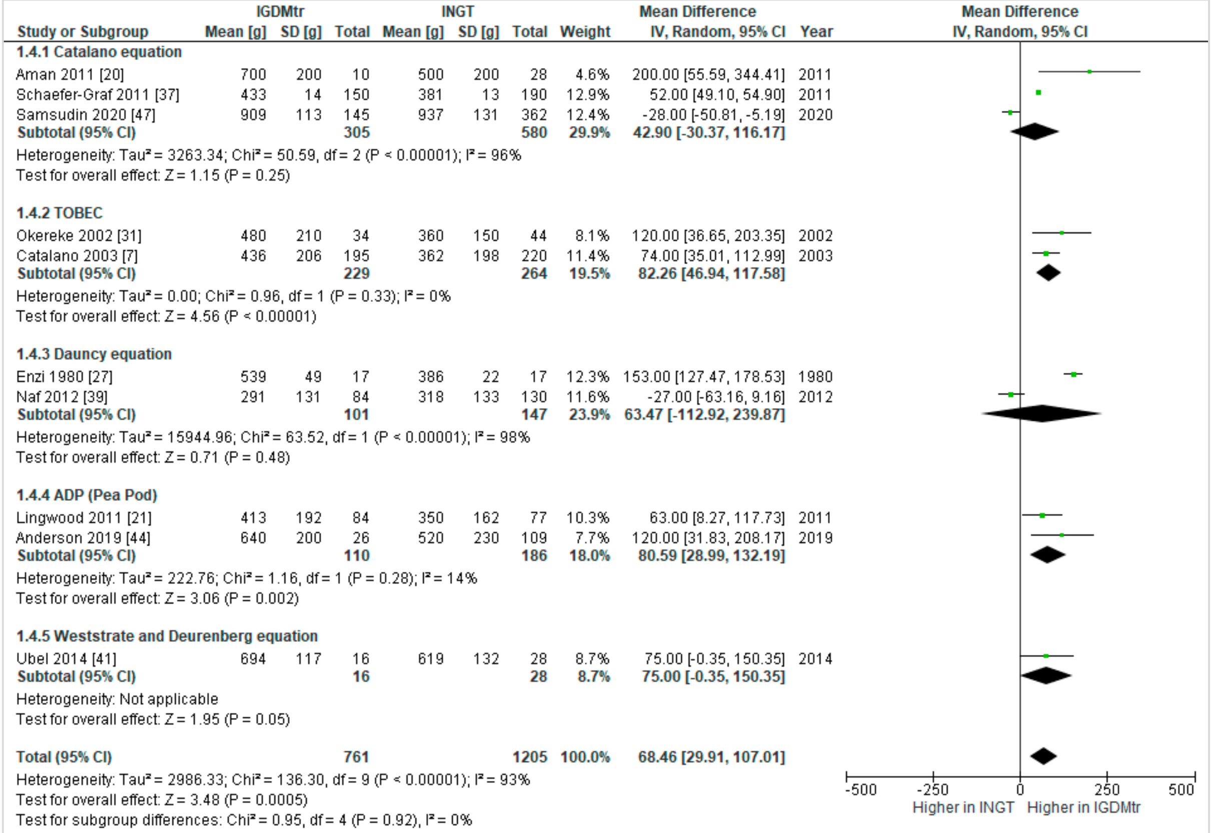

Figure S4. Forest plot comparing fat mass (g) in infants exposed to treated gestational diabetes mellitus (IGDMtr) and infants exposed to normal glucose tolerance (INGT) by subgroup analysis of infant body composition assessment technique.

Study or Subgroup	IGDMtr Mean [%]	IGDMtr SD [%]	IGDMtr Total	INGT Mean [%]	INGT SD [%]	INGT Total	Weight	Mean Difference IV, Random, 95% CI	Year
**1.5.1 Catalano equation**									
Aman 2011 [20]	17	3.2	10	13.5	3.5	28	9.6%	3.50 [1.13, 5.87]	2011
Maple-Brown 2019 [45]	11.5	3.6	235	10.6	3.9	739	12.8%	0.90 [0.36, 1.44]	2019
**Subtotal (95% CI)**			**245**			**767**	**22.4%**	**1.93 [-0.56, 4.43]**	
Heterogeneity: Tau² = 2.61; Chi² = 4.40, df = 1 (P = 0.04); I² = 77%									
Test for overall effect: Z = 1.52 (P = 0.13)									
**1.5.2 TOBEC**									
Okereke 2002 [31]	13.2	4.3	34	10.5	3.8	44	10.7%	2.70 [0.87, 4.53]	2002
Catalano 2003 [7]	12.4	4.6	195	10.4	4.6	220	12.4%	2.00 [1.11, 2.89]	2003
**Subtotal (95% CI)**			**229**			**264**	**23.1%**	**2.13 [1.34, 2.93]**	
Heterogeneity: Tau² = 0.00; Chi² = 0.46, df = 1 (P = 0.50); I² = 0%									
Test for overall effect: Z = 5.24 (P < 0.00001)									
**1.5.3 Dauncy equation**									
Enzi 1980 [27]	17.1	1.7	17	12.2	0.5	17	12.4%	4.90 [4.06, 5.74]	1980
**Subtotal (95% CI)**			**17**			**17**	**12.4%**	**4.90 [4.06, 5.74]**	
Heterogeneity: Not applicable									
Test for overall effect: Z = 11.40 (P < 0.00001)									
**1.5.4 ADP (Pea Pod)**									
Lingwood 2011 [21]	12.1	4.3	84	10.1	4.1	77	11.8%	2.00 [0.70, 3.30]	2011
Au 2013 [22]	7.3	4.5	67	9.3	4.3	532	12.0%	-2.00 [-3.14, -0.86]	2013
Brumbaugh 2013 [40]	14.7	3	12	13.1	5	13	7.9%	1.60 [-1.60, 4.80]	2013
Anderson 2019 [44]	16.4	4.7	26	13.9	4.5	109	10.4%	2.50 [0.51, 4.49]	2019
**Subtotal (95% CI)**			**189**			**731**	**42.1%**	**0.93 [-1.61, 3.47]**	
Heterogeneity: Tau² = 5.72; Chi² = 27.58, df = 3 (P < 0.00001); I² = 89%									
Test for overall effect: Z = 0.72 (P = 0.47)									
**Total (95% CI)**			**680**			**1779**	**100.0%**	**1.98 [0.54, 3.42]**	
Heterogeneity: Tau² = 4.17; Chi² = 109.11, df = 8 (P < 0.00001); I² = 93%									
Test for overall effect: Z = 2.69 (P = 0.007)									
Test for subgroup differences: Chi² = 26.53, df = 3 (P < 0.00001), I² = 88.7%									

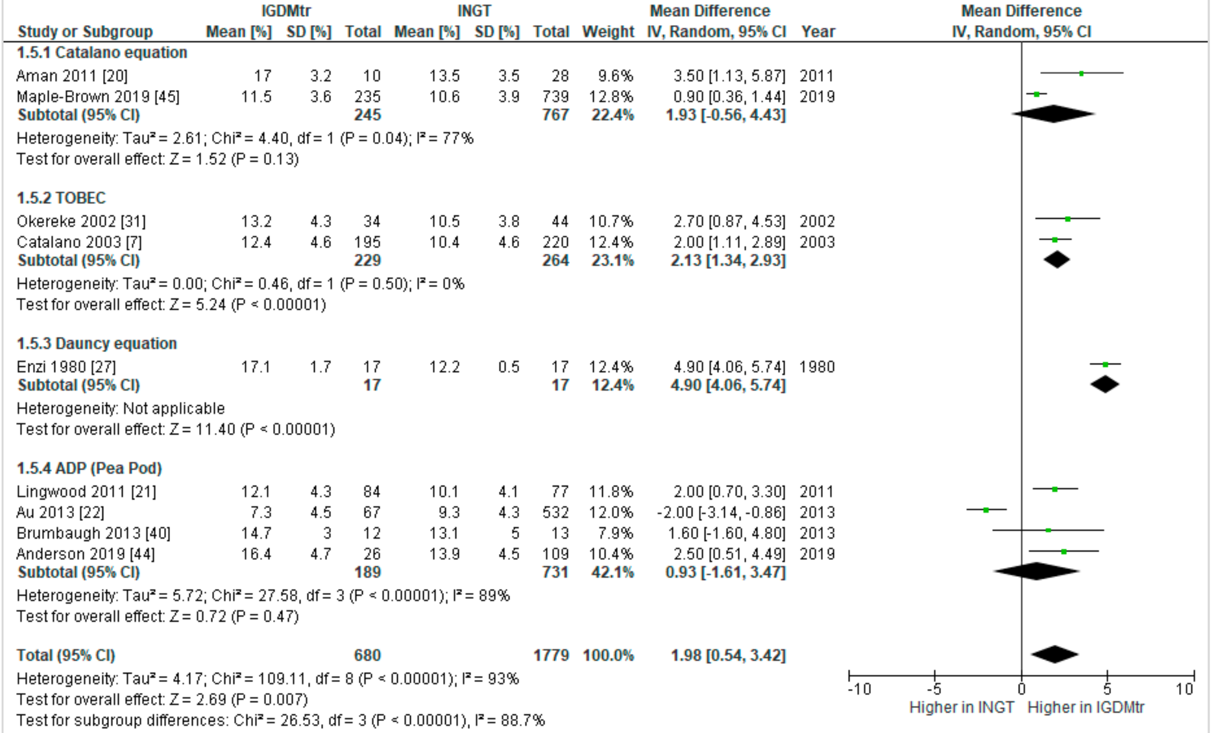

Figure S5. Forest plot comparing percent fat mass (%) in infants exposed to treated gestational diabetes mellitus (IGDM) and infants exposed to normal glucose tolerance (INGT) by subgroup analysis of infant body composition assessment technique.

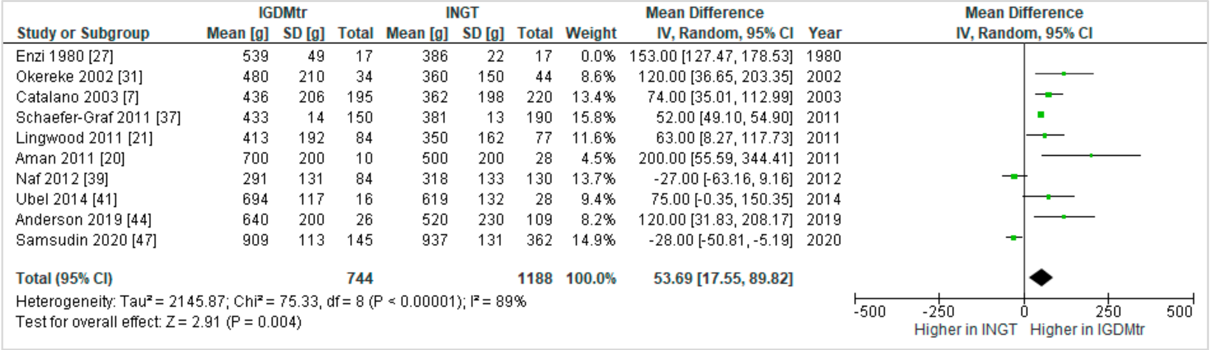

(a)

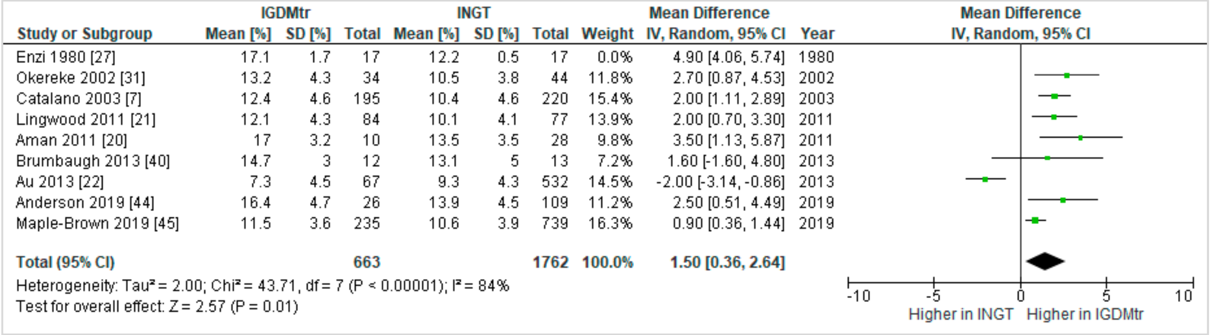

(b)

Figure S6. Forest plot comparing fat mass (a) and percent fat mass (b) in infants exposed to treated gestational diabetes mellitus (IGDM) and infants exposed to normal glucose tolerance (INGT) excluding the effect by the study (Enzi 1980) that used White's classification for diagnosis of gestational diabetes.

Reference

O'Sullivan, J.B.; Mahan, C.M. CRITERIA FOR THE ORAL GLUCOSE TOLERANCE TEST IN PREGNANCY. *Diabetes* **1964**, *13*, 278-285.
2. Stevenson, D.K.; Ochikubo, C.G.; Rodgers, P.A.; Kerner, J.A., Jr. Anthropometry and bilirubin production. *J Perinatol* **1991**, *11*, 340-342.
3. White, P. Classification of obstetric diabetes. *Am J Obstet Gynecol* **1978**, *130*, 228-230, doi:10.1016/0002-9378(78)90373-3.
4. Enzi, G.; Inelmen, E.M.; Caretta, F.; Villani, F.; Zanardo, V.; DeBiasi, F. Development of adipose tissue in newborns of gestational-diabetic and insulin-dependent diabetic mothers. *Diabetes* **1980**, *29*, 100-104, doi:10.2337/diab.29.2.100.
5. Group, N.D.D. Classification and Diagnosis of Diabetes Mellitus and Other Categories of Glucose Intolerance. *Diabetes* **1979**, *28*, 1039, doi:10.2337/diab.28.12.1039.
6. Catalano, P.M.; Thomas, A.; Huston-Presley, L.; Amini, S.B. Increased fetal adiposity: A very sensitive marker of abnormal in utero development. *American Journal of Obstetrics and Gynecology* **2003**, *189*, 1698-1704, doi:10.1016/s0002-9378(03)00828-7.
7. Naf, S.; Escote, X.; Yanez, R.E.; Ballesteros, M.; Simon, I.; Gil, P.; Megia, A.; Vendrell, J. Zinc-alpha2-glycoprotein is unrelated to gestational diabetes: anthropometric and metabolic determinants in pregnant women and their offspring. *PLoS One* **2012**, *7*, e47601, doi:10.1371/journal.pone.0047601.
8. Carpenter, M.W.; Coustan, D.R. Criteria for screening tests for gestational diabetes. *American Journal of Obstetrics and Gynecology* **1982**, *144*, 768-773, doi:https://doi.org/10.1016/0002-9378(82)90349-0.
9. Brumbaugh, D.E.; Tearse, P.; Cree-Green, M.; Fenton, L.Z.; Brown, M.; Scherzinger, A.; Reynolds, R.; Alston, M.; Hoffman, C.; Pan, Z.X., et al. Intrahepatic Fat Is Increased in the Neonatal Offspring of Obese Women with Gestational Diabetes. *Journal of Pediatrics* **2013**, *162*, 930-U977, doi:10.1016/j.jpeds.2012.11.017.
10. Hill, J.C.; Krishnaveni, G.V.; Annamma, I.; Leary, S.D.; Fall, C.H.D. Glucose tolerance in pregnancy in South India: relationships to neonatal anthropometry. *Acta Obstetricia Et Gynecologica Scandinavica* **2005**, *84*, 159-165, doi:10.1111/j.0001-6349.2005.00670.x.
11. Lain, K.Y.; Garabedian, M.J.; Daftary, A.; Jeyabalan, A. Neonatal adiposity following maternal treatment of gestational diabetes with glyburide compared with insulin. *American Journal of Obstetrics and Gynecology* **2009**, *200*, doi:10.1016/j.ajog.2009.02.038.
12. Okereke, N.C.; Uvena-Celebrezze, J.; Hutson-Presley, L.; Amini, S.B.; Catalano, P.M. The effect of gender and gestational diabetes mellitus on cord leptin concentration. *Am J Obstet Gynecol* **2002**, *187*, 798-803, doi:10.1067/mob.2002.125887.
13. Vohr, B.R.; McGarvey, S.T.; Coll, C.G. Effects of maternal gestational diabetes and adiposity on neonatal adiposity and blood pressure. *Diabetes Care* **1995**, *18*, 467-475, doi:10.2337/diacare.18.4.467.
14. N, F. Summary and Recommendations of the Second International Workshop-Conference on Gestational Diabetes Mellitus. *Diabetes* **1985**, *34*, 123-126, doi:10.2337/diab.34.2.S123.
15. Hollingsworth, D.R.; Vaucher, Y.; Yamamoto, T.R. Diabetes in pregnancy in Mexican Americans. *Diabetes Care* **1991**, *14*, 695-705, doi:10.2337/diacare.14.7.695.
16. Court, D.J.; Mann, S.L.; Stone, P.R.; Goldsbury, S.M.; Dixon-McIvor, D.; Baker, J.R. Comparison of glucose polymer and glucose for screening and tolerance tests in pregnancy. *Obstet Gynecol* **1985**, *66*, 491-499.
17. Simmons, D.; Robertson, S. Influence of maternal insulin treatment on the infants of women with gestational diabetes. *Diabet Med* **1997**, *14*, 762-765, doi:10.1002/(SICI)1096-9136(199709)14:9<762::AID-DIA485>3.0.CO;2-C.
18. Fuhrmann, K. Gestational Diabetes, Significance of Risk Factors and Results of a Follow-up Study 8 Years After Delivery. In *Gestational Diabetes*, Weiss, P.A.M., Coustan, D.R., Eds. Springer Vienna: Vienna, 1988; 10.1007/978-3-7091-8925-2_6pp. 93-98.
19. Plagemann, A.; Harder, T.; Kohlhoff, R.; Rohde, W.; Dorner, G. Overweight and obesity in infants of mothers with long-term insulin-dependent diabetes or gestational diabetes. *Int J Obes Relat Metab Disord* **1997**, *21*, 451-456.
20. Lind, T.; Phillips, P.R. Influence of pregnancy on the 75-g OGTT. A prospective multicenter study. The Diabetic Pregnancy Study Group of the European Association for the Study of Diabetes. *Diabetes* **1991**, *40 Suppl 2*, 8-13, doi:10.2337/diab.40.2.s8.
21. Andersson-Hall, U.K.; Järvinen, E.A.J.; Bosaeus, M.H.; Gustavsson, C.E.; Hårsmar, E.J.; Niklasson, C.A.; Albertsson-Wikland, K.G.; Holmäng, A.B. Maternal obesity and gestational diabetes mellitus affect body composition through infancy: the PONCH study. *Pediatric Research* **2019**, *85*, 369-377, doi:10.1038/s41390-018-0248-9.
22. Hoffman, L.; Nolan, C.; Wilson, J.D.; Oats, J.J.N.; Simmons, D. Gestational diabetes mellitus - management guidelines: The Australasian Diabetes in Pregnancy Society. *Medical Journal of Australia* **1998**, *169*, 93-97, doi:10.5694/j.1326-5377.1998.tb140192.x.

23. Au, C.P.; Raynes-Greenow, C.H.; Turner, R.M.; Carberry, A.E.; Jeffery, H.E. Body Composition Is Normal in Term Infants Born to Mothers With Well-Controlled Gestational Diabetes Mellitus. *Diabetes Care* **2013**, *36*, 562-564, doi:10.2337/dc12-1557.
24. Lingwood, B.E.; Henry, A.M.; d'Emden, M.C.; Fullerton, A.M.; Mortimer, R.H.; Colditz, P.B.; Le Cao, K.A.; Callaway, L.K. Determinants of Body Fat in Infants of Women With Gestational Diabetes Mellitus Differ With Fetal Sex. *Diabetes Care* **2011**, *34*, 2581-2585, doi:10.2337/dc11-0728.
25. Ng, P.C.; Lee, C.H.; Lam, C.W.; Wong, E.; Chan, I.H.; Fok, T.F. Plasma ghrelin and resistin concentrations are suppressed in infants of insulin-dependent diabetic mothers. *J Clin Endocrinol Metab* **2004**, *89*, 5563-5568, doi:10.1210/jc.2004-0736.
26. Rowan, J.A.; Hague, W.M.; Gao, W.; Battin, M.R.; Moore, M.P.; Mi, G.T.I. Metformin versus insulin for the treatment of gestational diabetes. *N Engl J Med* **2008**, *358*, 2003-2015, doi:10.1056/NEJMoa0707193.
27. Metzger, B.E.; Coustan, D.R. Summary and recommendations of the Fourth International Workshop-Conference on Gestational Diabetes Mellitus. The Organizing Committee. *Diabetes Care* **1998**, *21 Suppl 2*, B161-167.
28. Landon, M.B.; Spong, C.Y.; Thom, E.; Carpenter, M.W.; Ramin, S.M.; Casey, B.; Wapner, R.J.; Varner, M.W.; Rouse, D.J.; Thorp, J.M., Jr., et al. A multicenter, randomized trial of treatment for mild gestational diabetes. *N Engl J Med* **2009**, *361*, 1339-1348, doi:10.1056/NEJMoa0902430.
29. Salim, R.; Hasanein, J.; Nachum, Z.; Shalev, E. Anthropometric parameters in infants of gestational diabetic women with strict glycemic control. *Obstet Gynecol* **2004**, *104*, 1021-1024, doi:10.1097/01.AOG.0000143821.00194.ad.
30. Association, A.D. Gestational Diabetes Mellitus. *Diabetes Care* **2004**, *27*, S88-S90, doi:10.2337/diacare.27.2007.s88.
31. Buhling, K.J.; Doll, I.; Siebert, G.; Catalano, P.M. Relationship between sonographically estimated fetal subcutaneous adipose tissue measurements and neonatal skinfold measurements. *Ultrasound Obstet Gynecol* **2012**, *39*, 558-562, doi:10.1002/uog.10092.
32. Schaefer-Graf, U.M.; Meitzner, K.; Ortega-Senovilla, H.; Graf, K.; Vetter, K.; Abou-Dakn, M.; Herrera, E. Differences in the implications of maternal lipids on fetal metabolism and growth between gestational diabetes mellitus and control pregnancies. *Diabet Med* **2011**, *28*, 1053-1059, doi:10.1111/j.1464-5491.2011.03346.x.
33. Coustan, D.R.; Lowe, L.P.; Metzger, B.E.; Dyer, A.R. The Hyperglycemia and Adverse Pregnancy Outcome (HAPO) study: paving the way for new diagnostic criteria for gestational diabetes mellitus. *American Journal of Obstetrics and Gynecology* **2010**, *202*, 654.e651-654.e656, doi:10.1016/j.ajog.2010.04.006.
34. Maple-Brown, L.; Lee, I.L.; Longmore, D.; Barzi, F.; Connors, C.; Boyle, J.A.; Moore, E.; Whitbread, C.; Kirkwood, M.; Graham, S., et al. Pregnancy And Neonatal Diabetes Outcomes in Remote Australia: the PANDORA study-an observational birth cohort. *Int J Epidemiol* **2019**, *48*, 307-318, doi:10.1093/ije/dyy245.
35. Mitanchez, D.; Jacqueminet, S.; Nizard, J.; Tanguy, M.L.; Ciangura, C.; Lacorte, J.M.; De Carne, C.; L'Helias, L.F.; Chavatte-Palmer, P.; Charles, M.A., et al. Effect of maternal obesity on birthweight and neonatal fat mass: A prospective clinical trial. *Plos One* **2017**, *12*, doi:10.1371/journal.pone.0181307.
36. Prentice, P.M.; Olga, L.; Petry, C.J.; Simmons, D.; Murphy, H.R.; Hughes, I.A.; Acerini, C.L.; Ong, K.K.; Dunger, D.B. Reduced size at birth and persisting reductions in adiposity in recent, compared with earlier, cohorts of infants born to mothers with gestational diabetes mellitus. *Diabetologia* **2019**, *62*, 1977-1987, doi:10.1007/s00125-019-4970-6.
37. Uebel, K.; Pusch, K.; Gedrich, K.; Schneider, K.T.; Hauner, H.; Bader, B.L. Effect of maternal obesity with and without gestational diabetes on offspring subcutaneous and preperitoneal adipose tissue development from birth up to year-1. *BMC Pregnancy Childbirth* **2014**, *14*, 138, doi:10.1186/1471-2393-14-138.
38. World Health Organization. Diagnostic criteria and classification of hyperglycaemia first detected in pregnancy: a World Health Organization Guideline. *Diabetes Res Clin Pract* **2014**, *103*, 341-363, doi:10.1016/j.diabres.2013.10.012.
39. Kara, M.; Orbak, Z.; Doneray, H.; Ozkan, B.; Akcay, F. The Relationship Between Skinfold Thickness and Leptin, Ghrelin, Adiponectin, and Resistin Levels in Infants of Diabetic Mothers. *Fetal Pediatr Pathol* **2017**, *36*, 1-7, doi:10.1080/15513815.2016.1217960.
